# Design, synthesis, and evaluation of a novel PET imaging agent targeting lipofuscin in senescent cells†

**DOI:** 10.1039/d2ra04535d

**Published:** 2022-09-15

**Authors:** Diana Brickute, Cen Chen, Marta Braga, Chris Barnes, Ning Wang, Louis Allott, Eric O. Aboagye

**Affiliations:** Comprehensive Cancer Imaging Centre, Imperial College London, Hammersmith Hospital Du Cane Road London W12 0NN UK eric.aboagye@imperial.ac.uk; Positron Emission Tomography Research Centre, Faculty of Health Sciences, University of Hull Cottingham Road, Kingston upon Hull HU6 7RX UK louis.allott@hull.ac.uk; Department of Biomedical Sciences, Faculty of Health Sciences, University of Hull Cottingham Road, Kingston upon Hull HU6 7RX UK

## Abstract

Promoting a senescent phenotype to suppress tumour progression may present an alternative strategy for treating cancer and encourages the development of positron emission tomography (PET) imaging biomarkers for assessing response to treatment. The accumulation of lipofuscin deposits in senescent cells is visualised using the pathology stain Sudan Black B (SBB) which is an emerging biomarker of senescence. We describe the design, synthesis and evaluation of [^18^F]fluoroethyltriazole-SBB ([^18^F]FET-SBB), a fluorine-18 radiolabelled derivative of SBB. The *in vitro* uptake of [^18^F]FET-SBB in a senescent cell line corelated with lipofuscin deposits; *in vivo* PET imaging and metabolite analysis confirm a favourable pharmacokinetic and metabolic profile for further studies of *in vivo* models of senescence.

## Abstract 2

Lipofuscin deposits in senescence cells are visualised using the pathology stain Sudan Black B (SBB), an emerging biomarker of senescence. This work develops and evaluates a fluorine-18 derivative for positron emission tomography (PET) imaging.

## Graphical abstract

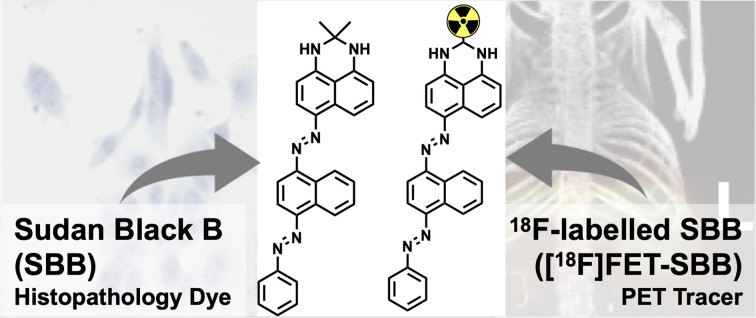

## Introduction

Senescence describes a state where cells irreversibly enter cell cycle arrest driven by a variety of stimuli, including telomere shortening, DNA damage, oncogenic activation and extrinsic stress.^1,2^ The biological process of cellular senescence is fundamental to normal cell development, embryonic development, wound healing, and aging. Oncogene-induced senescence (OIS) and therapy-induced senescence (TIS) play important roles in cancer biology, and in response to stress stimuli, can act as a potent barrier against tumour progression through intrinsic and extrinsic mechanisms. Pro-senescence therapies could be an alternative strategy for treating cancer.^3^ There have been several drugs showing potential effects on triggering cellular senescence in tumours. Palbociclib is a selective CDK4/6 inhibitor and has been approved for treating patients with ER-positive, HER2-negative advanced breast cancer.^4^ Palbociclib inhibits the activity of the CDK4/6-cyclin D1 complex, resulting in reduced phosphorylation of retinoblastoma (Rb) protein which in turn attenuates the release of E2F transcription factors from the pRb-E2F complex, thereby leading to the G0/G1 phase arrest and block of S-phase entry.^5^

A selective inhibitor of aurora A kinase (MLN8054) has been studied for the treatment of several cancer types including colorectal, breast, bladder and pancreatic tumours. MLN8054 treatment results in G2/M accumulation, spindle defects and anti-proliferative effects in multiple cultured human tumour cell lines. Although senescence is classically defined as an irreversible cell cycle arrest in G0/G1 phase, recently, several studies have shown that the senescence program can be also launched after G2 arrest.^6–8^

Given that senescence can be activated in response to various cancer therapies, there is a growing need for robust biomarkers, including molecular imaging biomarkers. Positron emission tomography (PET) imaging of senescent cells offers a sensitive approach to imaging the phenotype. An increase in senescence-associated beta-galactosidase (SA-β-GAL) activity is an important biomarker for senescence, and the PET radiotracer [^18^F]FPyGal was developed to image this pathway and has entered clinical evaluation; SA-β-GAL does not increase in all senescent cells and, false positives/negatives may occur.^9^

Another hallmark of senescence is the accumulation of lipofuscin, a waste product of aggregated proteins, lipids and metals produced by the senescence process.^10–12^ Although the mechanism for the formation of lipofuscin has not been identified, reactive oxygen species (ROS) produced during senescence is thought to have an influence. Increased ROS levels result in the carbonylation of several protein residues such as proline, threonine, lysine and arginine, in the presence of metal.^13^ These carbonyl residues further convert to Schiff bases *via* reacting with amino groups and promote protein aggregation. The insoluble lipofuscin is eventually formed by cross linking the protein aggregates with sugars and lipids.^13^

**Fig. 1:**
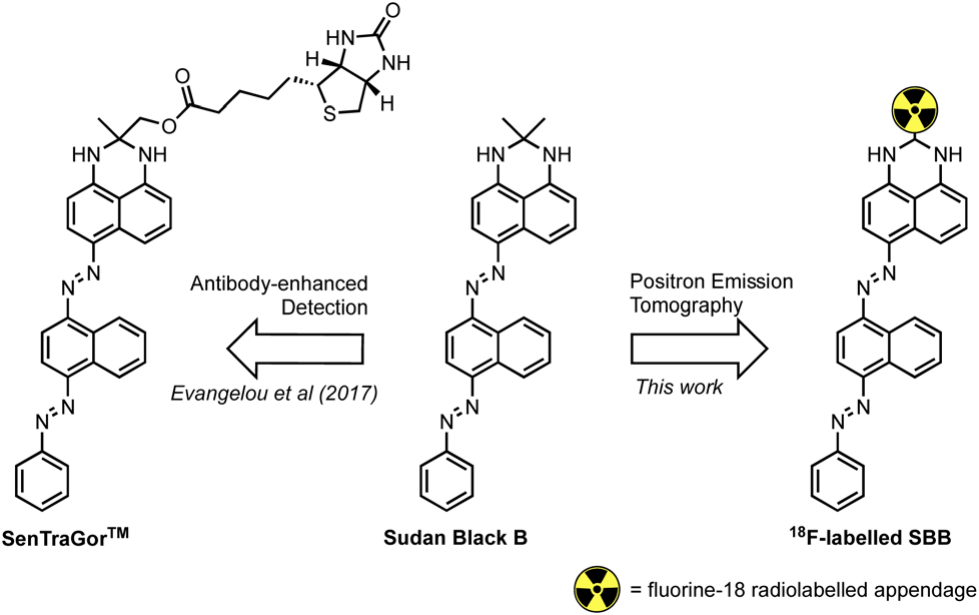
The structure of Sudan Black B (SBB), the SenTraGor™ derivative and a generic structure proposed for this work.

Georgakopoulou *et al.* (2013) reported the specific recognition of senescent cells using the histopathology dye Sudan Black B (SBB) which stained lipofuscin granules.^11^ To improve the sensitivity and specificity of SBB, Evangelou *et al.* (2017) developed a biotin-linked SBB derivative for the antibody-enhanced detection of lipofuscin;^14^ the reliability of this technology prompted commercialisation of the molecule for the recognition of senescent cells and is sold under the trademark name of SenTraGor™ (Fig. 1).

Encouraged by this work, we aimed to develop the first radiolabelled derivative of SBB to utilise the unparalleled sensitivity of PET for the detection and quantification of lipofuscin in senescent cells.

The key aims of this study were:

(1) Design, synthesise and radiolabel a derivative of SBB with fluorine-18.

(2) Evaluate the ability of non-radioactive and radioactive SBB derivatives to accumulate in lipofuscin deposits.

(3) Determine *in vivo* pharmacokinetics (PK), by PET imaging, in non-tumour bearing mice and evaluate *in vivo* metabolism.

Long term, we intended to identify a lead candidate for further preclinical evaluation using *in vivo* senescence models, and ultimately clinical translation.

## Results and discussion

### Chemistry

We hypothesised that a fluorine-18 radiolabelled analogue of SBB would allow for *in vitro* and *in vivo* detection of lipofuscin by PET. The SBB derivative developed by Evangelou *et al.* (2017) was substituted with a biotin linker at the *gem*-dimethyl position of the molecule (Fig. 1).^14,15^ Since, the molecule retained the ability to bind lipofuscin, we inferred that this position of the molecule may tolerate bulky groups and be a suitable location to incorporate a fluorine-18 radiolabelled appendage.

**Scheme 1:**
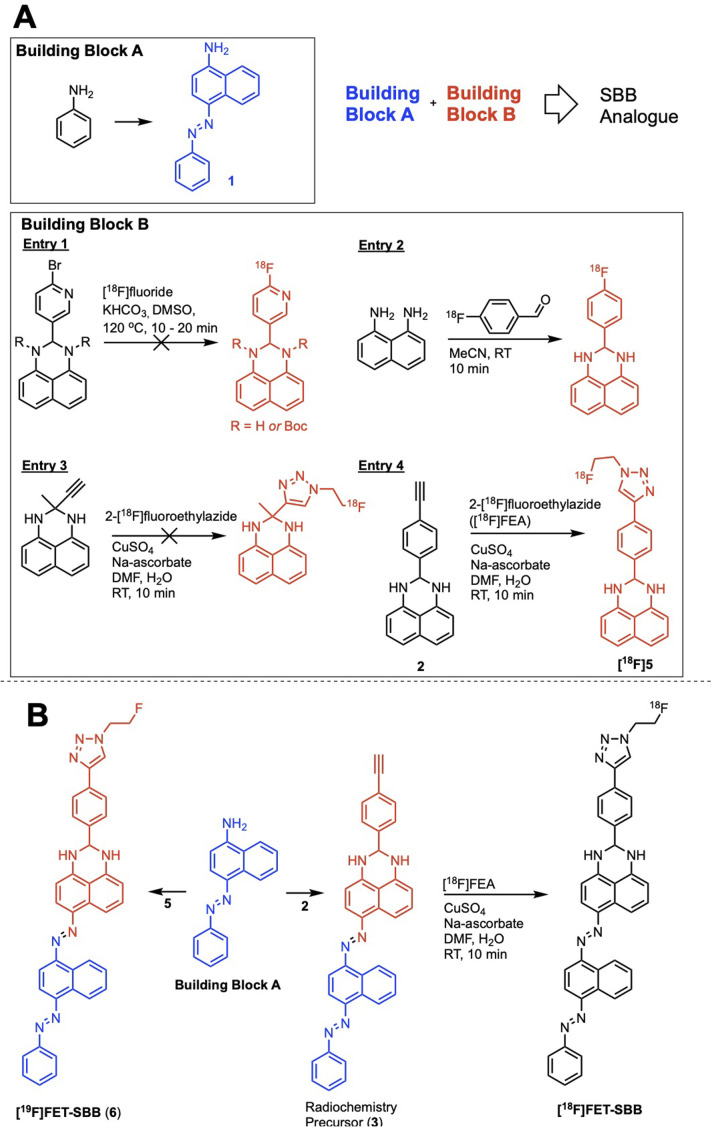
(A) The “building block” strategy used to assemble an SBB analogue and screen radiolabelling methods; (B) synthetic route to the radiochemistry precursor (3), reference standard [^19^F]FET-SBB (6) and the fluorine-18 radiolabelled [^18^F]FET-SBB.

To simplify the chemistry, we split the molecule into “building blocks” A and B (Scheme 1A). All SBB analogues were produced using the same common building block A, synthesised by the diazotisation of aniline to form an azo-bond with naphthalen-1-amine (1) in an excellent yield of 91%. Structural modifications to furnish radiochemistry precursors and reference standards were made to building block B. In addition, this strategy allowed for simple model compounds (building block B) to be screened for radiolabelling conditions to reduce the resources and time required to produce an expansive library of SBB analogues. Promising strategies would progress into SBB analogues by linking building block A to B *via* an azo-bond.

A range of radiolabelling chemistries were screened including direct ^18^F-fluorination of a pyridine moiety *via* nucleophilic aromatic substitution (S_N_Ar) chemistry with [^18^F]fluoride (Scheme 1A, entry 1); prosthetic group strategies using 4-[^18^F]fluorobenzaldehyde were also investigated (Scheme 1A, entry 2). Direct fluorination strategies were unsuccessful, likely due the presence of two secondary amines in the molecule. The amines were protected with *tert*-butyl-carbamate (Boc), however the high temperatures required for S_N_Ar radiochemistry resulted in rapid deprotection and prevented significant fluorination. A prosthetic group approach using 1,8-diaminonapthalene as a model precursor reacted rapidly with 4-[^18^F]fluorobenzaldehyde ([^18^F]FBA); however, synthesis of the corresponding SBB radiochemistry precursor was unsuccessful, likely due to diazotisation of the 1,8-diamino groups.

Copper-catalysed azide–alkyne cycloaddition (CuAAC) “click” chemistry was investigated as a chemoselective, mild and rapid method for radiolabelling alkyne-containing molecules with the small 2-[^18^F]fluoroethylazide ([^18^F]FEA) prosthetic group. A simple precursor (Scheme 1A, entry 3) was synthesised but no reaction with [^18^F]FEA was observed; potential chelation between this molecule and the copper catalyst (CuSO_4_·5H_2_O) may have inhibited the “click” reaction as chelation between similar structures has been reported in the literature.^16^ An aromatic spacer group was introduced into the molecule to overcome potential chelation of copper (Scheme 1A, entry 4). The precursor was synthesised by simply reacting 4-ethynylbenzaldehyde with 1,8-diaminonapthalene to produce 2 in excellent yield (77%), which radiolabelled efficiently with [^18^F]FEA (data not shown). The reference compound (6) was synthesised by CuAAC “click” chemistry between fluoroethylazide and 4-ethynylbenzaldehyde to form a 4-(1-(2-fluoroethyl)-1*H*-1,2,3-triazol-4-yl)benzaldehyde (4); subsequent reaction with 1,8-diaminonapthalene produced 5 (yield: 38%). With a suitable radiolabelling strategy, building block A and building block B (precursor and reference material) in hand, the full SBB analogue was assembled to produce precursor 5 and reference standard 6 (FET-SBB) as shown in Scheme 1B. Full synthetic schemes are shown in the ESI.†

The precursor (3) and reference material (6, FET-SBB) were purified by flash column chromatography and were synthesised in 2 and 4 steps, respectively. All compounds were characterised by ^1^H-NMR, ^13^C-NMR and ^19^F-NMR (ESI, Fig. S1–S15†).

### Radiochemistry

The radiolabelled analogue [^18^F]FET-SBB was synthesised by adapting our previously published automated procedure for CuAAC “click” radiolabelling using the [^18^F]FEA prosthetic group on the GE FASTLab™ platform, following Scheme 1B.^17^ The crude reaction mixture containing [^18^F]FET-SBB was purified by semi-preparative HPLC and reformulated into ethanol using tC18 solid-phase extraction (SPE) cartridges. The desired radiolabelled molecule was synthesised in a 5% RCY, non-decay corrected within 120 min. The molar activity (*A*_m_) of [^18^F]FET-SBB was 6.9 GBq μmol^−1^ and radiochemical purity (RCP) was >95%. The log *D*_7.5_ was determined to be 1.57 ± 0.18 (*n* = 3) using the shake-flask method.

#### Establishing an *in vitro* senescence model

Palbociclib and MLN8054 were expected to induce senescence to generate *in vitro* cancer cell models with which to evaluate FET-SBB. The lipofuscin content of the cell lines was determined using fluorescence microscopy by taking advantage of lipofuscin's autofluorescence. Palbociclib was shown to effectively inhibit *in vitro* cell growth of MCF-7 and T47D. Moreover, in response to palbociclib, MCF-7 cells displayed several senescence-associated features in a dose and time-dependent manner, as shown by an irreversible G0/G1 phase arrest, enlarged cell size and flattened cell shape, increased p21 expression and elevated SA-β-gal activity (ESI, Fig. S20†). In T47D cells, elevated SA-β-gal activity was not observed despite a senescence-like cell morphology. Collectively, palbociclib appeared to induce cellular senescence in MCF-7 and T47D cells.

MLN8054 effectively inhibited cell proliferation in HCT116 cells and caused a G2/M phase arrest. Cells under MLN8054-mediated G2/M cycle arrest were shown to re-enter cycling phase following withdrawal of drug. This indicated that the G2/M arrest induced by MLN8054 was a reversible – quiescent state – and not senescence. MCF-7 cells showed no response to MLN8054 under the indicated treatment conditions.

**Fig. 2:**
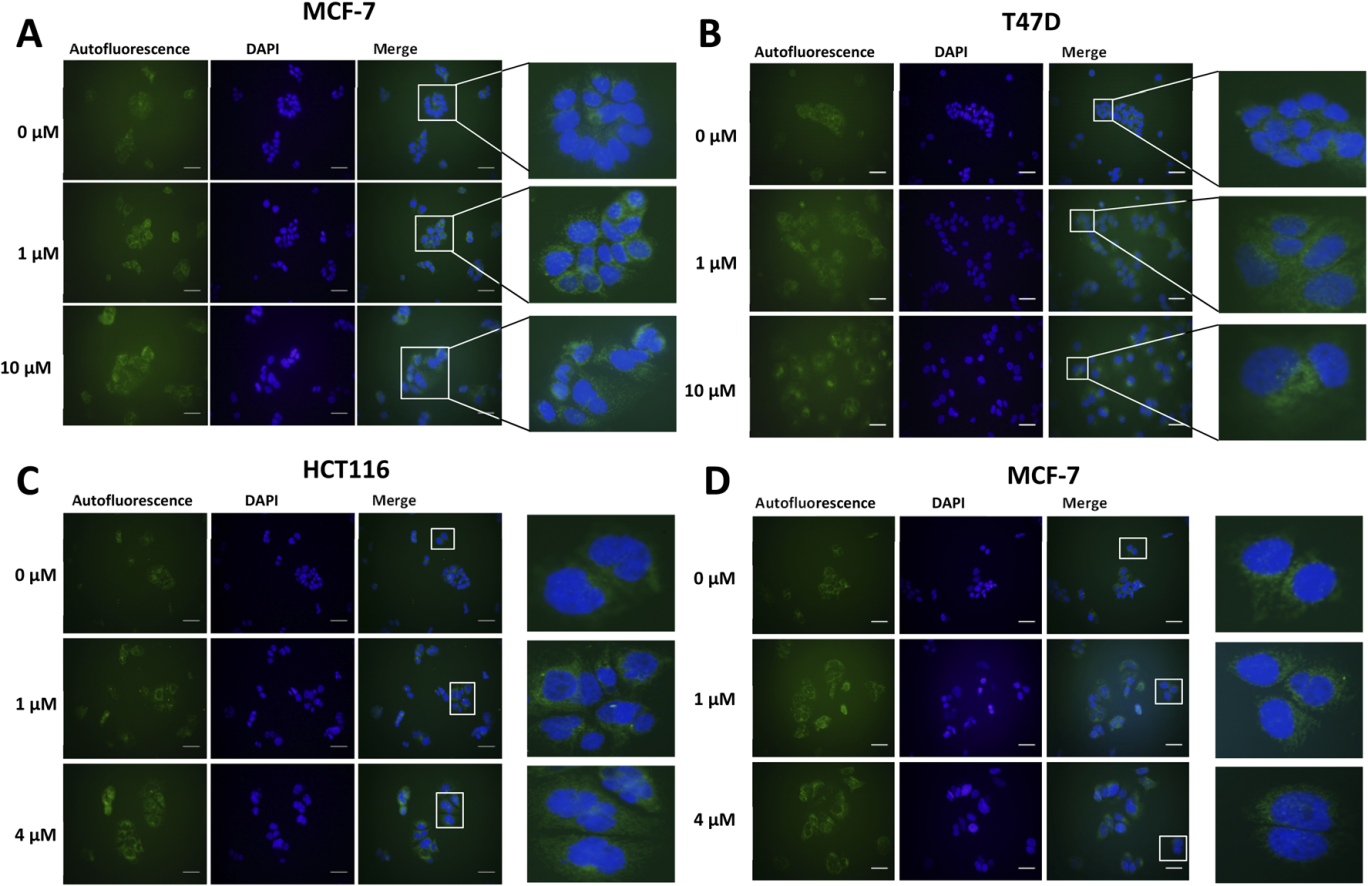
Representative images of autofluorescence of lipofuscin in MCF-7 (A) and T47D (B) cells following palbociclib treatment, and in HCT116 (C) and MCF-7 (D) cells following MLN8054 treatment. Briefly, cells were treated with palbociclib or MLN8054 at indicated concentrations for 72 h and then seeded at each appropriate density on pre-coated circular coverslips and placed within 12-well plates. After 24 h, cells were fixed with 4% PFA for 5 min and incubated with DAPI solution overnight. Images were obtained on a standard fluorescence microscopy under ×400 magnification. Scale bar = 50 μm. The green colour represents lipofuscin's autofluorescence and the blue colour represents cell nuclei stained by DAPI.

Lipofuscin accumulation in response to palbociclib and MLN8054 was determined using the autofluorescence of lipofuscin, visualised as granules by fluorescence microscopy (Fig. 2). The optimal fluorescent channels for detection are FITC (*λ*_ex_ = 488 nm and *λ*_em_ = 509 nm) or TRICT channel (*λ*_ex_ = 535 nm and *λ*_em_ = 572 nm). Palbociclib exhibits intrinsic fluorescence and therefore it was important rule out any overlap of its excitation and emission spectra with the autofluorescence of lipofuscin. The photochemical properties of palbociclib were determined by UV-Vis (*λ*_ex_) and fluorescence spectroscopy (*λ*_em_). Palbociclib showed an optimal photochemical characteristic (*λ*_ex_ = 365 nm and *λ*_em_ = 660 nm) that did not overlap with lipofuscin (spectra shown in ESI†).

An increased intensity of autofluorescence produced by lipofuscin in MCF-7 and T47D cells following palbociclib treatment was observed; MLN8054 treatment did not result in the formation of lipofuscin, shown by the absence of green fluorescence in HCT116 and MCF-7 cells. With a validated *in vitro* model of senescence in hand, we were able to evaluate [^19^F]FET-SBB by *in vitro* cell staining and [^18^F]FET-SBB by *in vitro* cell uptake.

##### *In vitro* evaluation of [^19^F]FET-SBB

**Fig. 3:**
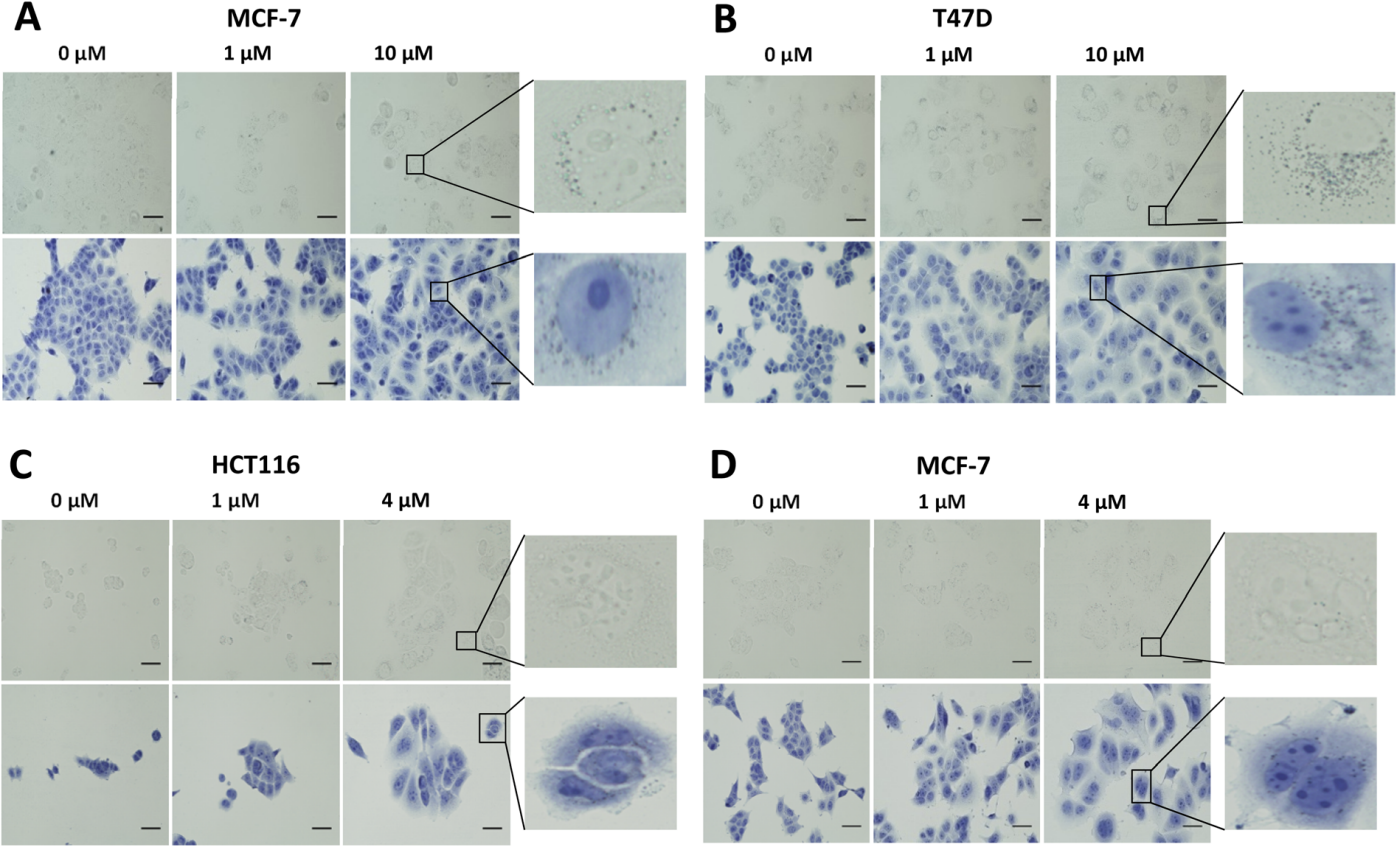
Representative images of [^19^F]FET-SBB staining in MCF-7 (A) and T47D (B) cells following palbociclib treatment, and in HCT116 (C) and MCF-7 (D) cells following MLN8054 treatment. Briefly, cells were treated with palbociclib or MLN8054 at indicated concentrations for 72 h and re-plated in 8-well chamber slides at each appropriate density. After 24 h, cells were fixed in ice-cold methanol for 5 min and incubated with [^19^F]FET-SBB solution for 10 min. Cells were quickly washed in 50% (v/v) ethanol, followed by PBS several times. Cells were finally counterstained in Hematoxylin Solution, Gill No. 3, for 1 min and rinsed in running tap water. Images were obtained on a standard microscopy under ×400 magnification. Scale bar = 50 μm. FET-SBB perinuclear accumulation as dark blue–black granules. Upper images, [^19^F]FET-SBB stain alone; Lower images: [^19^F]FET-SBB stain followed by hematoxylin counterstain.

Non-radioactive [^19^F]FET-SBB was used as an *in vitro* stain to determine if the compound bound to lipofuscin deposits (Fig. 3). MCF-7 and T47D cells, following palbociclib treatment, contained visible dark-blue perinuclear and cytoplasmic aggregates in a dose-dependent manner, stained by [^19^F]FET-SBB as dark-blue granules, representing the accumulation of lipofuscin. MLN8054 treatment, however, did not lead to similar aggregates stained by [^19^F]FET-SBB. These data are consistent with the pattern of lipofuscin autofluorescence, suggesting that the specific staining of lipofuscin by [^19^F]FET-SBB in palbociclib-induced senescent cells is similar to SBB, extensively reported in the literature. The mechanism of how [^19^F]FET-SBB binds to lipofuscin remains unclear; it is likely to work in the same way as SBB *via* interacting with the lipid compartment of lipofuscin enabled by its high lipophilicity.^15^ The promising specificity of [^19^F]FET-SBB to measure lipofuscin warranted further investigation of the radiolabelled derivative [^18^F]FET-SBB as a imaging probe for senescence-associated lipofuscin accumulation.

##### *In vitro* evaluation of [^18^F]FET-SBB

**Fig. 4:**
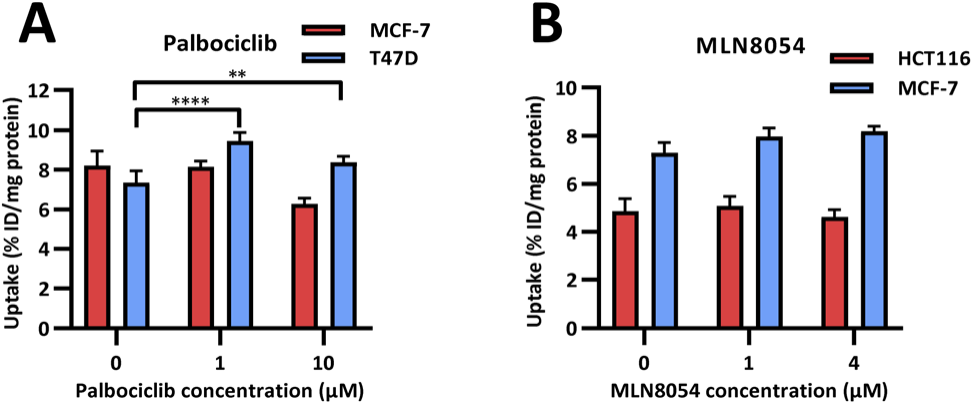
*In vitro* uptake of [^18^F]FET-SBB in cancer cells pre-treated with palbociclib (A) and MLN8054 (B).

Radioactive PET uptake experiments were conducted using [^18^F]FET-SBB in the same *in vitro* model of senescence previously described (Fig. 4). Tracer retention was significantly increased in T47D cells in response to palbociclib, from 7.40 ± 0.58 %ID/mg protein in control cells to 9.44 ± 0.43 %ID/mg protein at 1 μM palbociclib and 8.38 ± 0.30 %ID/mg protein at 10 μM palbociclib. Unexpectedly, [^18^F]FET-SBB uptake decreased from 8.21 ± 0.73% ID/mg protein in untreated MCF-7 cells to 6.27 ± 0.30 %ID/mg protein in palbociclib treated MCF-7 cells (10 μM).

One explanation for the decreased uptake of [^18^F]FET-SBB in palbociclib treated MCF-7 cells may be the possibility that palbociclib facilitates the expression of efflux transporters, which in turn rapidly pump out [^18^F]FET-SBB in MCF-7 cells. One of the limitations in the use of radioactive imaging agents is that the tracer is susceptible to efflux by members of the ATP binding cassette transporter proteins (ABC transporters). Although there is no direct evidence highlighting the regulatory effect of palbociclib on the expression or function of ABC transporters, palbociclib has been found to be a substrate for efflux proteins P-gp and breast cancer resistance protein (BCRP) at the blood–brain barrier.^18^

An alternative explanation is that palbociclib-mediated autophagy might partially contribute to the decreased tracer uptake in MCF-7 cells. In addition to senescence, palbociclib at a high dose (10 μM) also triggered cell autophagy in MCF-7 cells, as shown by increased expression of LC3B-II, which is a standard marker for autophagosomes (ESI, Fig. S20†). It has been reported that the expression level of P-gp is positively correlated with LC3B in tumour samples from colorectal cancer patients, strongly indicating that autophagy is related to the upregulation of efflux transporters.^19^

In accordance with [^19^F]FET-SBB staining, MLN8054 did not cause changes in the levels of [^18^F]FET-SBB uptake in HCT116 or MCF-7 cells despite a high uptake overall.

##### *In vivo* PET imaging

**Fig. 5:**
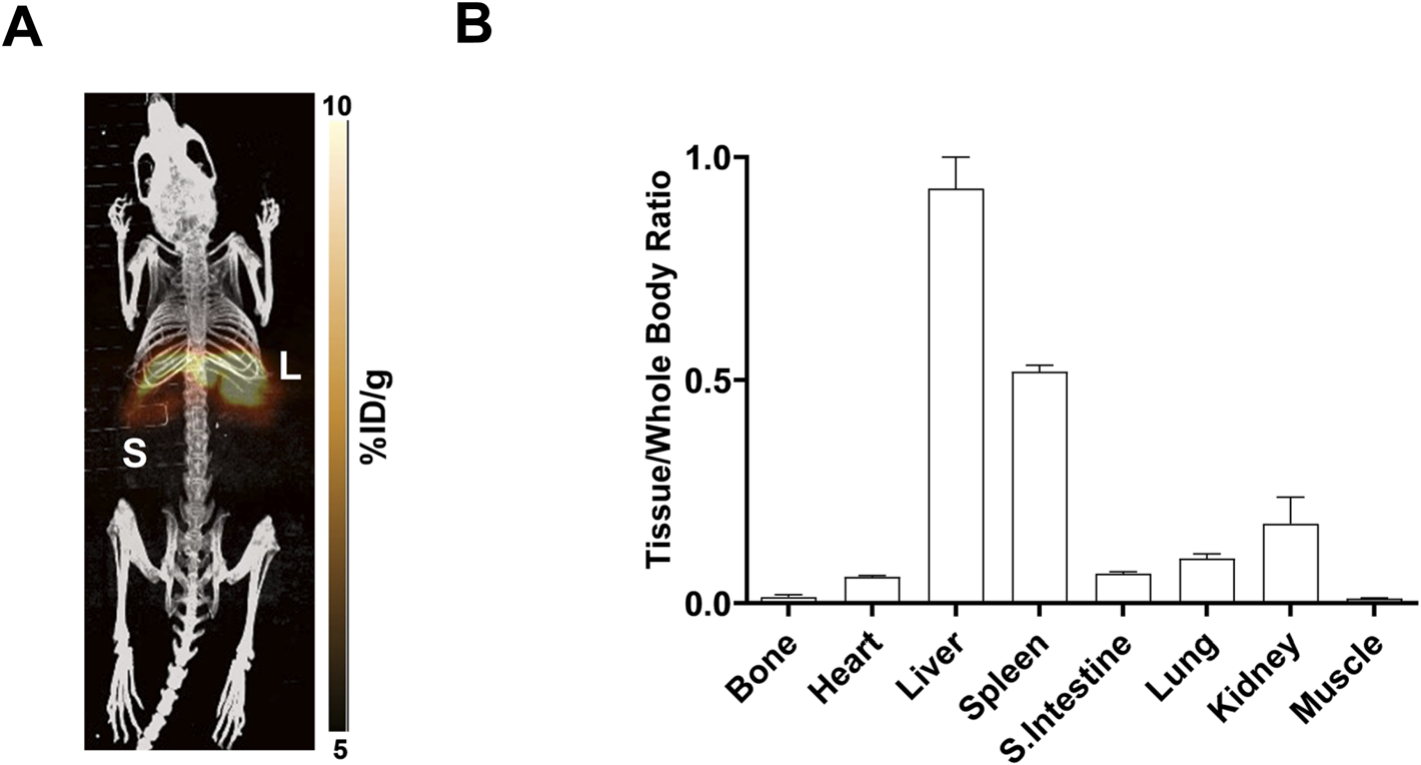
*In vivo* PET imaging of [^18^F]FET-SBB. (A) Representative maximum intensity projection image of [^18^F]FET-SBB PET scan (30–60 min) after injection of 2 MBq *via* the tail vein; (B) radioactive concentration of [^18^F]FET-SBB in key organs determined by region-of-interest analysis of the summed 30–60 min frames; tissue concentration was normalised to muscle uptake. L = liver, S = spleen.

Dynamic PET imaging was performed in non-tumour bearing mice (*n* = 4) to determine the distribution of [^18^F]FET-SBB *in vivo* (Fig. 5). The liver exhibits the highest signal in the PET images (summed frames 30–60 min post-injection, Fig. 5A), followed by the spleen, which was corroborated by region-of-interest analysis of the radioactive concentration (Fig. 5B).

**Fig. 6:**
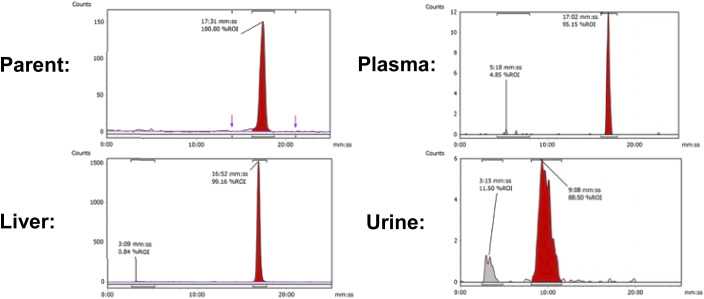
Representative radio-chromatograms of parent [^18^F]FET-SBB (*t*_R_ = 17:31 min:s) and metabolites extracted from liver, plasma and urine at 60 min p.i.; efficiencies of radioactivity extraction from plasma and liver were 77.6 ± 6.9% and 76.9 ± 5.5% of total radioactivity, respectively.

Uptake in the kidney was also relatively high, indicating some level of urinary excretion. The background radioactive accumulation in all the other organs was low, which may result in advantageous tumour imaging contrast in non-hepatic lesions. To better understand the chemical form of the radioactivity accumulated in the liver, we performed metabolite analysis (Fig. 6). The *in vivo* metabolites of [^18^F]FET-SBB in the liver, plasma and urine were determined by HPLC at 60 min post-injection. The parent radiotracer remained largely unmetabolised (>95%) in the liver and plasma, which is advantageous for PET imaging at late time points if target accumulation is slow; only two polar metabolites were identified in urine, however the quantity of radioactivity was very low (Fig. 6).

## Conclusions

We successfully synthesised a fluorine-containing derivative of SBB and evaluated its accumulation in lipofuscin granules by microscopy (cell staining with [^19^F]FET-SBB) and *in vitro* radioactive uptake [^19^F]FET-SBB accumulated in senescent cells in parallel with lipofuscin content, determined by lipofuscin autofluorescence. The radioactive [^18^F]FET-SBB followed the same trend in T47D cells with palbociclib-induced senescence; however, an unexpected decrease in uptake was observed in MCF-7 cells with palbociclib-induced senescence. Regardless, the absence of any trend in MLN8054 treated cells, where no lipofuscin was detected by autofluorescence, gives encouragement that [^18^F]FET-SBB may, to some degree, trace lipofuscin deposits; although our data is statistically significant, it may not be biologically/clinically relevant, which brings into question the sensitivity of [^18^F]FET-SBB, in its current form, for imaging lipofuscin deposits. The pharmacokinetic profile of [^18^F]FET-SBB was determined *in vivo* by PET alongside radioactive metabolite analysis, which showed extensive liver accumulation and largely intact parent radiotracer at 60 min p.i. and encourages further evaluation of [^18^F]FET-SBB in animals bearing senescent tissues. The log *D*_7.5_ of [^18^F]FET-SBB suggested that it is highly lipophilic. Lipophilic tracers tend to show off-target binding interactions; thus this was a concern. Contrary to the general trend for such compounds, however, [^18^F]FET-SBB showed low background localisation except in liver and urine suggesting no lipophilicity dependent affinity for neutral fats and lipids. Furthermore, a fundamental challenge relates to detection of senescent cells that constitute a smaller population of overall tumour cell population; though future treatments to induce senescence should increase the phenotype. In this regard, low background uptake of [^18^F]FET-SBB may aid improved detection.

## Experimental

### Materials and methods

Anhydrous solvents and reagents were purchased from Sigma Aldrich (Gillingham, UK) and were used without additional purification. Flash column chromatography purification was performed on silica gel (Merck Kieselgel 60 F_254_ 320–400 mesh). Thin Layer Chromatography (TLC) was performed on Merck aluminium-backed plates pre-coated with silica (0.2 mm, 60 F_254_) which were visualised by quenching of ultraviolet fluorescence (*λ* = 254 and 365 nm). ^1^H-NMR, ^13^C-NMR and ^19^F-NMR was obtained using a Bruker AV-400 spectrometer at a frequency of 400, 101 and 376 MHz, respectively. Chemical shifts (*δ*) are given in parts per million (ppm) and referenced to the appropriate residual solvent peaks. Signals are assigned as s, d, t, dt, m and br for singlet, doublet, triplet, double triplet, multiplet and broad respectively. Mass spectrometry was performed by the Mass Spectrometry Facility of the Chemistry Department of Imperial College London. Determination of log *D*_7.5_ was performed by the shake-flask method following a published procedure.^20^

### Chemistry

#### (*E*)-4-(Phenyldiazenyl)naphthalen-1-amine (1)

This compound was prepared according to the experimental procedure described in the patent WO2018002614A1, upon reaction of aniline with 1-naphtylamine to provide pure 1 as a red solid in 91% yield. ^1^H NMR (400 MHz, Chloroform-*d*) *δ* 9.08 (d, *J* = 9.3 Hz, 1H), 8.01 (d, *J* = 7.1 Hz, 2H), 7.94 (d, *J* = 8.2 Hz, 1H), 7.81 (d, *J* = 8.5 Hz, 1H), 7.72–7.62 (m, 1H), 7.60–7.49 (m, 3H), 7.44 (t, *J* = 7.3 Hz, 1H), 6.81 (d, *J* = 8.2 Hz, 1H), 4.59 (s, 2H). ^13^C NMR (101 MHz, Chloroform-*d*) *δ* 153.66, 146.34, 140.55, 133.27, 129.89, 129.15, 127.20, 125.47, 124.25, 122.78, 122.63, 120.72, 113.98, 109.24. HRMS (ESI) = 248.1188 (M + H)^+^. Calc. for C_16_H_14_N_3_: 248.1188.

#### 2-(4-Ethynylphenyl)-2,3-dihydro-1*H*-perimidine (2)

To a solution of 1,8-diaminonaphthalene (965 mg, 6.11 mmol) in absolute EtOH (15 mL) was added 4-ethynylbenzaldehyde (874 mg, 6.72 mmol). The reaction mixture was stirred at RT for 16 h. The resulting precipitate was filtered under vacuum, washed with cold absolute EtOH and air dried. The product (beige solid, 1.27 g, 77%) was used without further purification. ^1^H NMR (400 MHz, Chloroform-*d*) *δ* 7.66–7.55 (m, 4H), 7.34–7.22 (m, 4H), 6.56 (dd, *J* = 6.7, 1.6 Hz, 2H), 5.49 (s, 1H), 4.51 (s, 2H), 3.17 (s, 1H). ^13^C NMR (101 MHz, Chloroform-*d*) *δ* 141.92, 140.79, 134.99, 132.71, 128.06, 127.02, 123.56, 118.21, 113.55, 106.12, 83.23, 78.24, 68.12. HRMS (ESI) = 271.1230 (M + H)^+^. Calc. for C_19_H_15_N_2_: 271.1235.

#### 2-(4-Ethynylphenyl)-6-((*E*)-(4-((*E*)-phenyldiazenyl)naphthalen-1-yl)di-azenyl)-2,3-dihydro-1*H*-perimidine (3)

(*E*)-4-(Phenyldia-zenyl)naphthalen-1-amine 1 (128 mg, 0.52 mmol) was dissolved in DMF (2 mL), followed by addition of H_2_O (2 mL) and HCl (10 N, 0.4 mL). The resulting mixture was cooled to 0 °C and NaNO_2_ (35.88 mg, 0.52 mmol) in water (1 mL) was added dropwise. Reaction was stirred for 2 h at 0 °C, after which time, the resulting diazonium salt was added dropwise under vigorous stirring to a solution of perimidine 2 (140 mg, 0.52 mmol) in EtOH (5 mL) and DMF (1 mL) at 0 °C, the reaction was stirred at 0 °C for 1 h and then at RT for 1.5 h. A saturated solution of NaHCO_3_ was added to neutralise the reaction mixture, which resulted in formation of dark precipitate. The solution was left to stand at 0 °C for another hour before filtration under vacuum. The product was washed with H_2_O and air dried. The crude product (232 mg, 85%) gave 95% purity by HPLC and therefore no further purification was performed. ^1^H NMR (400 MHz, DMSO-*d*_6_) *δ* 9.30–8.79 (m, 2H), 8.36 (s, 1H), 8.29–8.16 (m, 2H), 8.14–8.06 (m, 2H), 8.01 (q, *J* = 8.4 Hz, 2H), 7.82 (td, *J* = 6.8, 6.3, 3.6 Hz, 2H), 7.74–7.51 (m, 7H), 7.45 (dd, *J* = 8.4, 7.5 Hz, 1H), 7.29 (s, 1H), 6.77–6.59 (m, 2H), 5.73 (s, 1H), 4.25 (s, 1H). ^13^C NMR (101 MHz, DMSO-*d*_6_) *δ* 152.81, 149.91, 148.38, 146.02, 142.85, 142.20, 138.69, 133.45, 131.98, 131.90, 131.82, 131.56, 131.05, 130.10, 129.59, 129.28, 127.87, 127.67, 127.08, 123.79, 123.06, 122.96, 122.07, 118.53, 112.74, 111.42, 110.78, 110.65, 105.87, 105.34, 83.22, 81.33, 65.20. HRMS (ESI) = 529.2134 (M + H)^+^. Calc. for C_35_H_25_N_6_: 529.2141.

#### 4-(1-(2-Fluoroethyl)-1*H*-1,2,3-triazol-4-yl)benzaldehyde (4)

To a solution of 4-ethynylbenzaldehyde (174.2 mg, 1.34 mmol) in DMF (1 mL) was added a mixture of copper (ii) sulfate pentahydrate (67.4 mg, 0.27 mmol) and sodium ascorbate (106.9 mg, 0.54 mmol) in *t*BuOH : H_2_O (1 : 1, 2 mL) at RT. The resulting solution was stirred at RT for 15 min before addition of 2-fluoroethylazide (1.34 mmol) in DMF (3.2 mL). The reaction mixture was stirred at RT for 16 h before quenching with water (30 mL). The crude product was extracted with DCM (3 × 25 mL) and washed with brine. After drying over Na_2_SO_4_, the solvent was removed under reduced pressure. The resulting material was purified by silica gel column chromatography (DCM : EtOAc, 40 : 1) to provide the product (110 mg, 38%) as a white solid. ^1^H NMR (400 MHz, Chloroform-*d*) *δ* 10.00 (s, 1H), 8.08–7.96 (m, 3H), 7.96–7.88 (m, 2H), 4.96–4.86 (m, 1H), 4.85–4.74 (m, 2H), 4.74–4.66 (m, 1H). ^13^C NMR (101 MHz, Chloroform-*d*) *δ* 191.78, 146.95, 136.26, 135.94, 130.47, 126.16, 121.95 (d, *J* = 2.1 Hz), 81.63 (d, *J* = 172.6 Hz), 50.85 (d, *J* = 20.4 Hz). ^19^F NMR (376 MHz, Chloroform-*d*) *δ* −221.51 (m). HRMS (ESI) = 220.0884 (M + H)^+^. Calc. for C_11_H_11_N_3_OF: 220.0886.

#### 2-(4-(1-(2-Fluoroethyl)-1*H*-1,2,3-triazol-4-yl)phenyl)-2,3-dihydro-1*H*-perimidine (5)

To a solution of 1,8-diaminonaphthalene (66 mg, 0.42 mmol) in absolute EtOH (10 mL) was added 4-(1-(2-fluoroethyl)-1*H*-1,2,3-triazol-4-yl)benzaldehyde (100 mg, 0.46 mmol). The reaction mixture was stirred at RT for 16 h. The resulting precipitate was filtered under vacuum, washed with cold absolute EtOH and purified by silica gel column chromatography (hexane : EtOAc, 1 : 2) to provide the product (120 mg, 80%) as a white solid. ^1^H NMR (400 MHz, Chloroform-*d*) *δ* 7.82 (d, *J* = 8.3 Hz, 3H), 7.61 (d, *J* = 8.2 Hz, 2H), 7.27–7.11 (m, 4H), 6.46 (dd, *J* = 6.8, 1.5 Hz, 2H), 5.41 (s, 1H), 4.89–4.77 (m, 1H), 4.74–4.65 (m, 2H), 4.65–4.57 (m, 1H), 4.50 (br s, 2H). ^13^C NMR (101 MHz, Chloroform-*d*) *δ* 147.75, 142.15, 140.14, 135.01, 131.81, 130.53, 128.60, 127.07, 126.24, 126.22, 121.00 (d, *J* = 2.0 Hz), 118.07, 113.58, 106.04, 81.75 (d, *J* = 172.5 Hz), 68.23, 50.80 (d, *J* = 20.5 Hz). ^19^F NMR (376 MHz, Chloroform-*d*) *δ* −221.39 (m). HRMS (ESI) = 360.1620 (M + H)^+^. Calc. for C_21_H_19_N_5_F: 360.1624.

#### 2-(4-(1-(2-Fluoroethyl)-1*H*-1,2,3-triazol-4-yl)phenyl)-6-((*E*)-(4-((*E*)-phenyldiazenyl)naphthalen-1-yl)diazenyl)-2,3-dihydro-1*H*-perimidine (6)

(*E*)-4-(Phenyldiazenyl)naphthalen-1-amine 1 (72 mg, 0.29 mmol) was dissolved in DMF (2 mL), followed by addition of H_2_O (2 mL) and HCl (10 N, 0.4 mL). The resulting mixture was cooled to 0 °C and NaNO_2_ (20 mg, 0.29 mmol) in water (1 mL) was added dropwise. Reaction was stirred for 2 h at 0 °C, after which time, the resulting diazonium salt was added dropwise under vigorous stirring to a solution of perimidine 5 (106 mg, 0.29 mmol) in EtOH (4 mL) and DMF (1.5 mL) at 0 °C, the reaction was stirred at 0 °C for 1 h and then at RT for another 1.5 h. Saturated solution of NaHCO_3_ was added to neutralise the reaction mixture, which resulted in formation of dark precipitate. The solution was left to stand at 0 °C for another hour before filtration under vacuum. The crude precipitate was washed with H_2_O and air dried before purification by silica gel column chromatography (hexane : EtOAc, 1 : 2) to provide the product (105 mg, 58%) as a black blue solid. ^1^H NMR (400 MHz, Chloroform-*d*) *δ* 9.19–9.08 (m, 1H), 9.08–9.00 (m, 1H), 8.51 (d, *J* = 8.5 Hz, 1H), 8.15 (d, *J* = 8.2 Hz, 1H), 8.12–8.06 (m, 2H), 8.05 (d, *J* = 8.3 Hz, 1H), 7.98 (d, *J* = 8.3 Hz, 1H), 7.90–7.80 (m, 3H), 7.78–7.68 (m, 2H), 7.64 (d, *J* = 8.2 Hz, 2H), 7.62–7.45 (m, 4H), 6.63 (dd, *J* = 15.5, 7.8 Hz, 2H), 5.56 (s, 1H), 5.13 (s, 1H), 4.86 (t, *J* = 4.6 Hz, 1H), 4.74 (t, *J* = 4.6 Hz, 1H), 4.69 (t, *J* = 4.6 Hz, 1H), 4.63 (t, *J* = 4.6 Hz, 1H, s, 1H). ^13^C NMR (101 MHz, Chloroform-*d*) *δ* 153.54, 150.36, 147.90, 147.55, 146.55, 142.02, 141.14, 139.26, 133.99, 132.61, 132.14, 132.06, 131.19, 129.30, 129.16, 128.52, 127.21, 126.90, 126.30, 124.17, 123.61, 123.40, 121.05, 116.59, 113.93, 112.78, 112.72, 112.26, 107.00, 106.18, 81.69 (d, *J* = 172.7 Hz), 68.02, 50.76 (d, *J* = 20.4 Hz). ^19^F NMR (376 MHz, Chloroform-*d*) *δ* −221.35 (m). HRMS (ESI) = 618.2535 (M + H)^+^. Calc. for C_37_H_29_N_9_F: 618.2530.

#### 2-Fluoroethylazide

2-Fluoroethyltosylate was prepared according to a literature procedure.^21^ To a solution of 2-fluoroethyl-4-toluenesulfonate (640 mg, 2.94 mmol) in anhydrous DMF (5 mL) was added sodium azide (572 mg, 8.8 mmol), and resulting solution was stirred at RT for 48 h. The reaction mixture was centrifuged, and the supernatant containing the title compound was used without isolation for subsequent reactions. **HAZARD**: 2-fluoroethylazide may be explosive when isolated, do not isolate this compound from solvent.

### Radiochemistry

No-carrier-added aqueous [^18^F]fluoride (typically 1 mL, 1.5–2.0 GBq) in enriched ^18^O water was delivered to the GE FASTlab™ radiosynthesis module and trapped on a Waters QMA-carbonate Sep-Pak SPE cartridge. The [^18^F]fluoride was eluted into the reactor vial using 800 μL of eluent (18 mg mL^−1^ Kryptofix 2.2.2 in 800 μL MeCN, 12 mg mL^−1^ KHCO_3_ in 200 μL H_2_O) using the syringe (position 3). The [^18^F]fluoride was dried under nitrogen and vacuum at 120 °C. The 2-azidoethyl-*p*-toluenesulfonate precursor (8 μL, 33 μmol) in anhydrous MeCN (1 mL) was added to the reactor which was sealed and heated at 80 °C for 10 min. The reactor was cooled and diluted in an off-board vial (40 mL) containing water (25 mL). An additional aliquot of water (2 mL) was used to rinse the reactor into the dilution vial using syringe (position 11). The diluted reaction mixture was passed through the tC18 plus SPE cartridge and the HLB plus SPE cartridge in series [pre-conditioned with 5 mL MeCN and 5 mL H_2_O]. The cartridges were washed with water (4 × 5 mL) and dried under nitrogen for 3 min. To the HLB plus SPE cartridge was carefully loaded 500 μL of a 1.1 mL solution of DMF and H_2_O (9% v/v) containing SBB precursor (16 mg), CuSO_4_ (15 mg, 60 μmol), sodium ascorbate (30 mg, 201.6 μmol) and bathophenanthroline disulfonic acid disodium salt trihydrate (BPDS) (20 mg, 34 μmol) was carefully loaded onto the HLB plus SPE cartridge using syringe 3. The “click” reaction was performed on the cartridge at ambient temperature for 10 min.^17^ The reaction mixture was eluted from the HLB plus SPE cartridge with MeCN (2.5 mL) into an off-board dilution vial (40 mL) containing HPLC mobile phase (8 mL, 85% MeCN in water). The semi-preparative HPLC loop (10 mL) was filled using a 5 mL syringe (position 24) and [^18^F] SBB was purified in 85% MeCN in water and at a flow rate of 4 mL min^−1^. A final solvent exchange step was performed by diluting the product peak cut (16 mL) in 11.2 mL of water to ensure a product solution containing 50 : 50 MeCN : H_2_O. The dilution was passed through a tC18 plus SPE cartridge. The cartridge was washed with water (2 × 5 mL) and dried under nitrogen for 3 min before the product was eluted in EtOH (1 mL).

### Cell culture

MCF-7 (breast carcinoma; ATCC), T47D (breast carcinoma; ATCC) and HCT116 (colorectal carcinoma; ATCC) were cultured in DMEM media (Sigma-Aldrich) supplemented with 10% foetal calf serum (FCS), 1% l-glutamine and 2% penicillin–streptomycin. All cell lines were cultured at 37 °C in a humidified atmosphere containing 5% CO_2_. All cell lines were routinely tested for mycoplasma and typically not passaged for longer than three months.

For determining the photochemical properties of palbociclib, the compound was freshly dissolved in DMSO at the indicated concentrations of 0.001, 0.01, 0.1, 1 and 2 μM. Samples were added to a 96-well dark plate in triplicate and assayed on a plate reader (Infinite 200 PRO, Tecan). The absorbance was plotted against the measured wavelength of 300 nm to 600 nm to obtain absorption spectra. Then the wavelength of maximum absorption was used as the excitation wavelength to obtain emission (fluorescence) spectra of palbociclib. The emission spectra show the relative fluorescence intensity, with the intensity plotted on the vertical axis *versus* wavelength on the horizontal axis.

### Determination of lipofuscin by autofluorescence

Cells were seeded and treated with palbociclib or MLN8054 at indicated concentrations for 72 h. At the end of treatment, cells were re-plated at each appropriate density on pre-coated circular coverslips placed within 12-well plates. After 24 h, cells were washed gently with PBS and fixed with 5% PFA for 5 min, in the meanwhile, a drop of DAPI solution was prepared on a rectangular coverslip. The coverslips with fixed cells were inverted allowing the cells facing down and laying over the DAPI solution. This attachment was left overnight in a dark at room temperature. On the next day, images were obtained on a standard fluorescence microscopy under ×400 magnification.

### Western blots

Membranes were probed using anti-p21 antibody (Waf1/Cip1, 2947, Cell Signalling, 1:1000) and anti-LC3B antibody (2775, Cell Signalling, 1:1000). Anti-β-actin (ab6276, Abcam, 1:10 000) was used as a loading control.

### Cell cycle analysis by flow cytometry (FACS)

Pre-treated cell pellets were collected, washed and centrifuged for 5 min at 1500×*g*. Cell pellets were re-suspended in 0.5 mL of ice-cold PBS and fixed in 70% ethanol by adding dropwise, then left in 4 °C fridge for at least 12 hours. On the day of FACS analysis, samples were centrifuged for 5 min at 1500×*g* and stained in PI staining solution containing 40 μg μL^−1^ propidium iodide (PI, P4170, Sigma-Aldrich) and 0.25 mg mL^−1^ RNase A (R5503, Sigma-Aldrich) for 30 min in 37 °C water bath in the dark. DNA cell cycle analysis was then performed on a BD FACSCanto flow cytometer (BD Bioscience). Data analysis was performed by using FlowJo.

### Senescence-associated β-galactosidase staining (SA-β-gal)

Palbociclib-induced senescence was examined by using the SA-β-gal staining kit (9860S, Cell Signalling) according to manufacturer's instructions. Cells were fixed for 15 min at room temperature in fixation solution and gently washed with PBS three times. Then cells were incubated with β-galactosidase staining solution in 37 °C incubator for approximately 20 hours. Images were obtained at ×400 magnification on a microscope (Olympus BX51) with a minimum of three fields for each condition. Quantification was done by counting the intensity of blue stained cells and normalising to the intensity of total cells of three independent fields by ImageJ.

### Staining of lipofuscin by [^19^F]FET-SBB

Cells were seeded and treated with palbociclib or MLN8054 at indicated concentrations for 72 h. At the end of treatment, cells were re-plated at each appropriate density on 8-well chamber slides and allowed to attach for 24 h. FET-SBB was freshly prepared in 70% ethanol at a concentration of 4 mg mL^−1^ and filtered through a 0.45 μm filter prior to use. Cells were fixed in ice-cold ethanol for 5 min and incubate with FET-SBB for 10–15 min. Then cells were quickly washed in 50% (v/v) ethanol, followed by PBS for several times. Cells were finally counterstained in Hematoxylin Solution, Gill No. 3 for 1 min and rinsed in running tap water. Images were obtained by standard microscopy under ×400 magnification.

### [^18^F]FET-SBB *in vitro* uptake

Cells were seeded and treated with palbociclib or MLN8054 at indicated concentrations for 72 h. At the end of treatment, cells were re-plated at each appropriate density in 6-well plates and allowed to attach for 24 h. On the day of uptake, cells were washed three times with pre-warmed PBS and incubated with 1 mL of fresh DMEM media containing approximately 0.74 MBq radiotracer ([^18^F]FET-SBB) for 1 h at 37 °C in a humidified condition of 5% CO_2_. Cells were then washed three times with ice-cold PBS and lysed in 1 mL of RIPA buffer for 10 min on ice. The radioactivity of 800 μL lysate from each sample was counted on a WIZARD2 Automatic Gamma Counter. Data were determined as a percentage of radioactivity incorporated into cells, normalised to total cellular protein as measured by BCA assay and expressed as %ID/mg protein.

### Statistical analysis

Data were expressed as mean values ± standard deviation (SD). Statistical tests were performed using Graphpad Prism software (v.8.3). Comparisons were considered statistically significant when *P* < 0.05.

### *In vivo* PET imaging

For PET imaging, BALB/c mice (Charles River UK Ltd, Margate, UK) were anesthetized and scanned on a dedicated small animal PET scanner (G4 Genesis, Sofie Biosciences, Culver City, CA, USA) following a bolus injection of 2 MBq of [^18^F]FET-SBB *via* a lateral tail vein cannula. Imaging was performed under 2% isoflurane/O_2_ anaesthesia. After tracer injection, emission scans were acquired in list-mode format (over 0–60 min – dynamic scans) to give decay-corrected values of radioactivity accumulation in tissues. The collected data were reconstructed with a 3-dimensional maximum likelihood estimation method 3D ML-EM (Sofie Biosciences). Cumulative images of the data were used for visualization of radiotracer uptake and to define tissue volumes of interest (VOIs) using Siemens Inveon Research Workplace software (Siemens Molecular Imaging, Inc. Knoxville, USA). Tissue radioactivity uptake values were normalized to injected dose and mouse weight. Whole-body ratios were determined by drawing a region of interest (ROI) segmenting the whole body *vs.* tissue ROI's.

### Metabolite analysis

In BALB/c mice at 60 min p.i. of [^18^F]FET-SBB (2 MBq), three key tissues (liver, urine and blood plasma) were analysed for radioactive metabolites by radio-HPLC (Agilent 1100 system) fitted with an in-line posiRAM metabolite detector (Lablogic, Sheffield, UK). An isocratic mobile phase (85% MeCN/15% H_2_O, 4 mL min^−1^) was used in conjunction with an Agilent Zorbax XDB C18 column (250 × 9.4 mm, 5 μ). The retention time of parent compound [^18^F]FET-SBB was determined by injecting a radioactive sample onto the metabolite radio-HPLC system. The liver was excised, and a portion homogenised in ice cold MeCN (1 mL) using a Precellys tissue homogeniser fitted with the Cryolys cooling module (Stretton Scientific Ltd, Derbyshire, UK). Solid tissues and protein were pelleted by centrifugation (13 000*g*, 5 min) and the supernatant was removed and filtered (0.22 μm syringe filter) before being diluted in H_2_O for radio-HPLC analysis. Urine was diluted in H_2_O and filtered prior to radio-HPLC analysis. Plasma was obtained from whole blood by centrifugation (2000*g*, 5 min) to separate the blood cells from the plasma; the plasma was removed and precipitated in ice cold MeCN (1 mL) and centrifuged (13 000 *g*, 5 min) to pellet the proteins. The supernatant was filtered (0.22 μm syringe filter) and diluted in H_2_O for radio-HPLC analysis. The HPLC injection loop (1 mL) was washed with mobile phase between each injection. The efficiency of extracting radioactivity from each plasma and liver sample was determined by counting the activity (counts per minute, CPM) in a small aliquot (20 μL) of the supernatant of a known volume and the whole protein pellet, in a λ-counter (PerkinElmer, Wizard2). The extraction efficiency of radioactivity from plasma and liver was 77.6 ± 6.9 and 76.9 ± 5.5% of total activity, respectively. Radio-HPLC chromatograms were integrated using Laura 6 software (Lablogic, Sheffield, UK).

## Ethical statement

All animal experiments were done by licensed investigators in accordance with the UK Home Office Guidance on the Operation of the Animal (Scientific Procedures) Act 1986 (HMSO, London, UK, 1990) and within guidelines set out by the UK National Cancer Research Institute Committee on Welfare of Animals in Cancer Research. To limit the use of animals in this study, only four non-tumour bearing mice were used for PET imaging and metabolite analysis.

## Conflicts of interest

There are no conflicts to declare.

## Abbreviations

*A*_m_
: Molar activity
BCA: Bicinchoninic acid assay
DCM: Dichloromethane
EtOAc: Ethyl acetate
RCP: Radiochemical purity
RCY: Radiochemical yield, non-decay corrected to start of radiosynthesis
RIPA: Radioimmunoprecipitation assay buffer
r.t: Room temperature
SD: Standard deviation
TEA: Triethylamine
TFA: Trifluoroacetic acid

## Supplementary Material

RA-012-D2RA04535D-s001
