# Supplementary material for: Design, synthesis, and evaluation of a novel PET imaging agent targeting lipofuscin in senescent cells
Source: RSC Adv. 2022 Sep 15;12(40):26372–81. doi: 10.1039/d2ra04535d (PMC9475417; doi:10.1039/d2ra04535d)
Supplement: RA-012-D2RA04535D-s001 [file RA-012-D2RA04535D-s001.pdf]

Electronic Supplementary Material (ESI) for RSC Advances.
This journal is © The Royal Society of Chemistry 2022

Design, synthesis, and evaluation of a novel PET imaging agent targeting lipofuscin in senescent cells

Diana Brickute,[a†] Cen Chen,[a†] Marta Braga,[a] Chris Barnes,[a] Ning Wang,[a] Louis Allott[b,c]* and Eric O. Aboagye[a]*

a. *Comprehensive Cancer Imaging Centre, Imperial College London, Hammersmith Hospital, Du Cane Road, London, UK, W12 0NN.*

b. *Positron Emission Tomography Research Centre, Faculty of Health Sciences, University of Hull, Cottingham Road, Kingston upon Hull, UK, HU6 7RX*

c. *Department of Biomedical Sciences, Faculty of Health Sciences, University of Hull, Cottingham Road, Kingston Upon Hull, UK, HU6 7RX*

† Equal contribution

* Corresponding author: Prof Eric O. Aboagye (eric.aboagye@imperial.ac.uk) and Dr Louis Allott (louis.allott@hull.ac.uk)

Supplementary Information

Contents

1.0 Materials and Methods **Page 1**
2.0 Synthesis **Page 2**
3.0 NMR Spectra **Page 3**
4.0 Radiochemistry **Page 11**
5.0 HPLC Chromatograms **Page 12**
6.0 Biological Evaluation **Page 14**
7.0 PET Imaging Data **Page 14**

1.0 Materials and Methods

Anhydrous solvents and reagents were purchased from Sigma Aldrich (Gillingham, UK) and were used without additional purification. Flash column chromatography purification was performed on silica gel (Merck Kieselgel 60 F_{254} 320-400 mesh). Thin Layer Chromatography (TLC) was performed on Merck aluminium-backed plates pre-coated with silica (0.2 mm, 60 F_{254}) which were visualised by quenching of ultraviolet fluorescence (λ = 254 and 365 nm). ^{1}H-NMR, ^{13}C-NMR and ^{19}F-NMR was obtained using a Bruker AV-400 spectrometer at a frequency of 400, 101 and 376 MHz, respectively. Chemical shifts (δ) are given in parts per million (ppm) and referenced to the appropriate residual solvent peaks. Signals are assigned as s, d, t, dt, m and br for singlet, doublet, triplet, double triplet, multiplet and broad respectively. Mass spectrometry was performed by the Mass Spectrometry Facility of the Chemistry Department of Imperial College London.

2.0 Synthesis

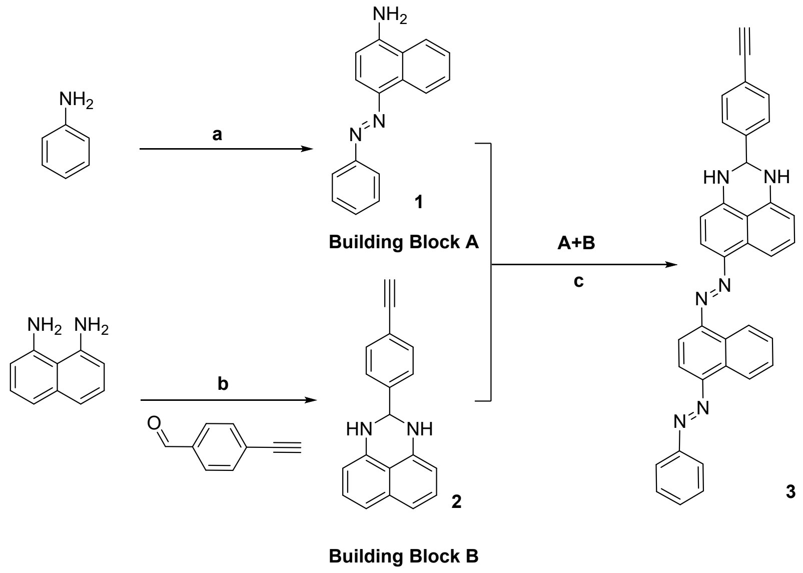

Scheme 1. Reagents and conditions: **a)** i) $NaNO_2$, 10N HCl, H_2O, 0 °C, 2h. ii) 1-naphthylamine, H_2O, EtOH, 10N HCl, 0 °C, 2h; then RT, 16h. **b)** EtOH, RT, overnight. **c)** i) $NaNO_2$, DMF, 10N HCl, H_2O, 0 °C, 2h. ii) perimidine **2**, EtOH, DMF, 0°C, 1h, then RT, 1.5h.

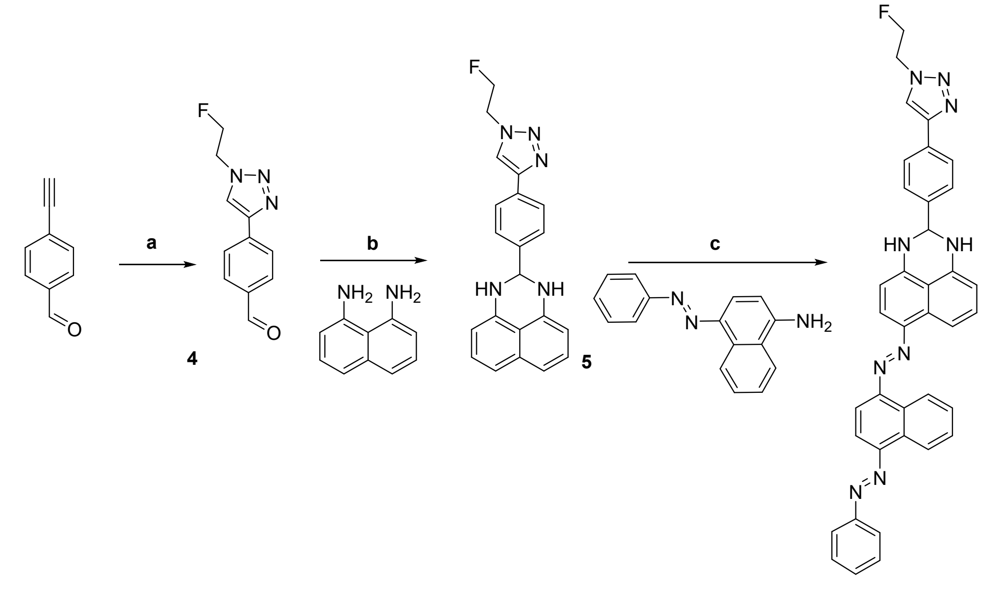

Scheme 2. Reagents and conditions: **a)** 2-fluoroethylazide, $CuSO_4{\cdot}5H_2O$, Na-ascorbate, *t*-BuOH:H_2O (1:1), DMF, RT, 16 h. **b)** EtOH, RT, 16 h. **c)** i) $NaNO_2$, DMF, 10N HCl, H_2O, 0 °C, 2 h. ii) perimidine **5**, EtOH, DMF, 0°C, 1 h; then RT, 1.5 h

3.0 NMR Spectra

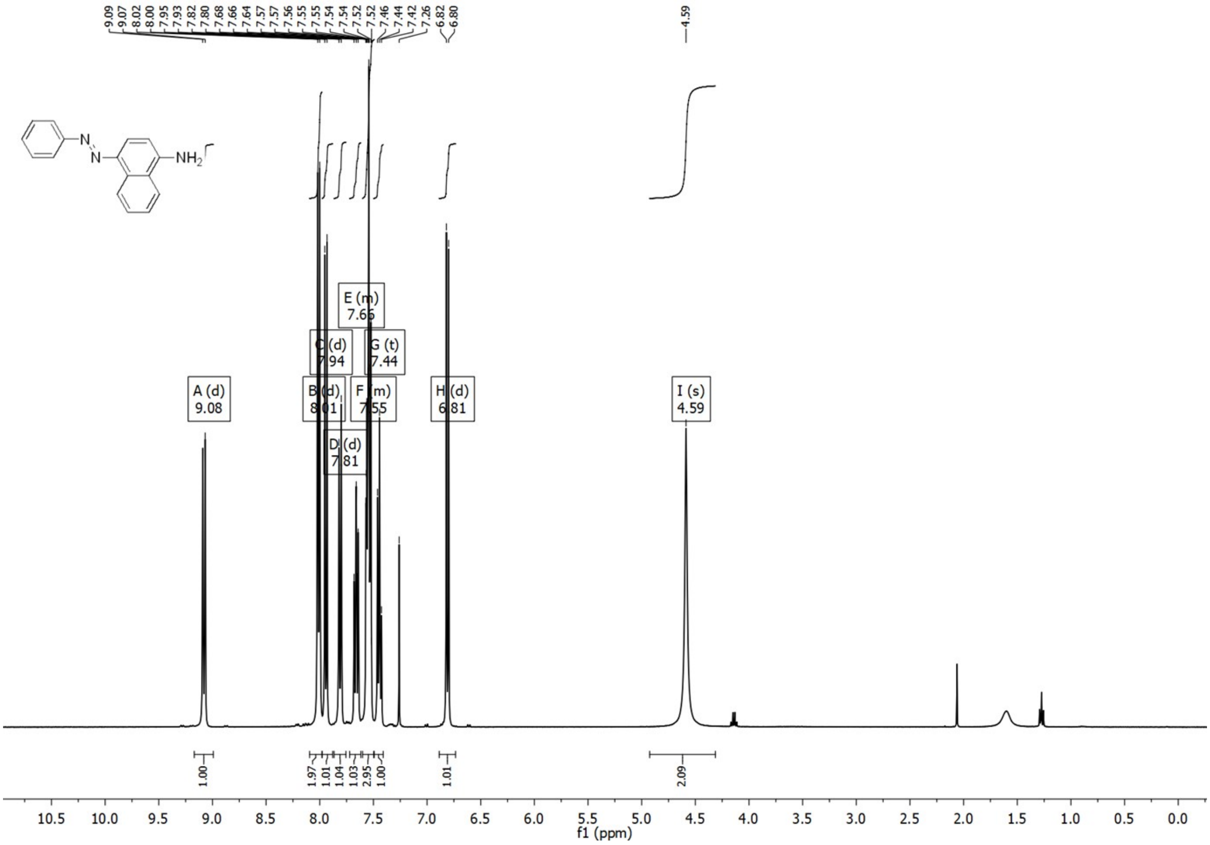

Figure 1. ^{1}H-NMR of (E)-4-(phenyldiazenyl)naphthalen-1-amine (**1**)

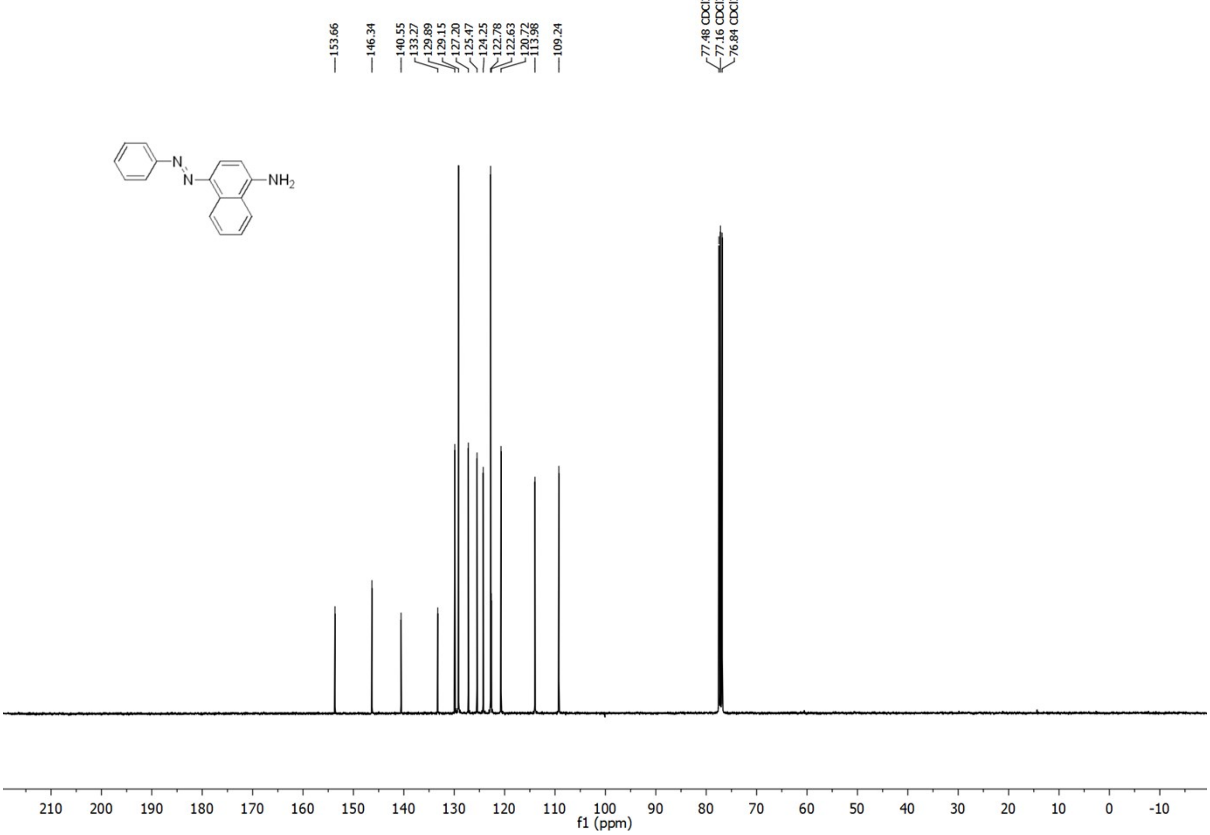

Figure 2. ^{13}C-NMR of (E)-4-(phenyldiazenyl)naphthalen-1-amine (**1**)

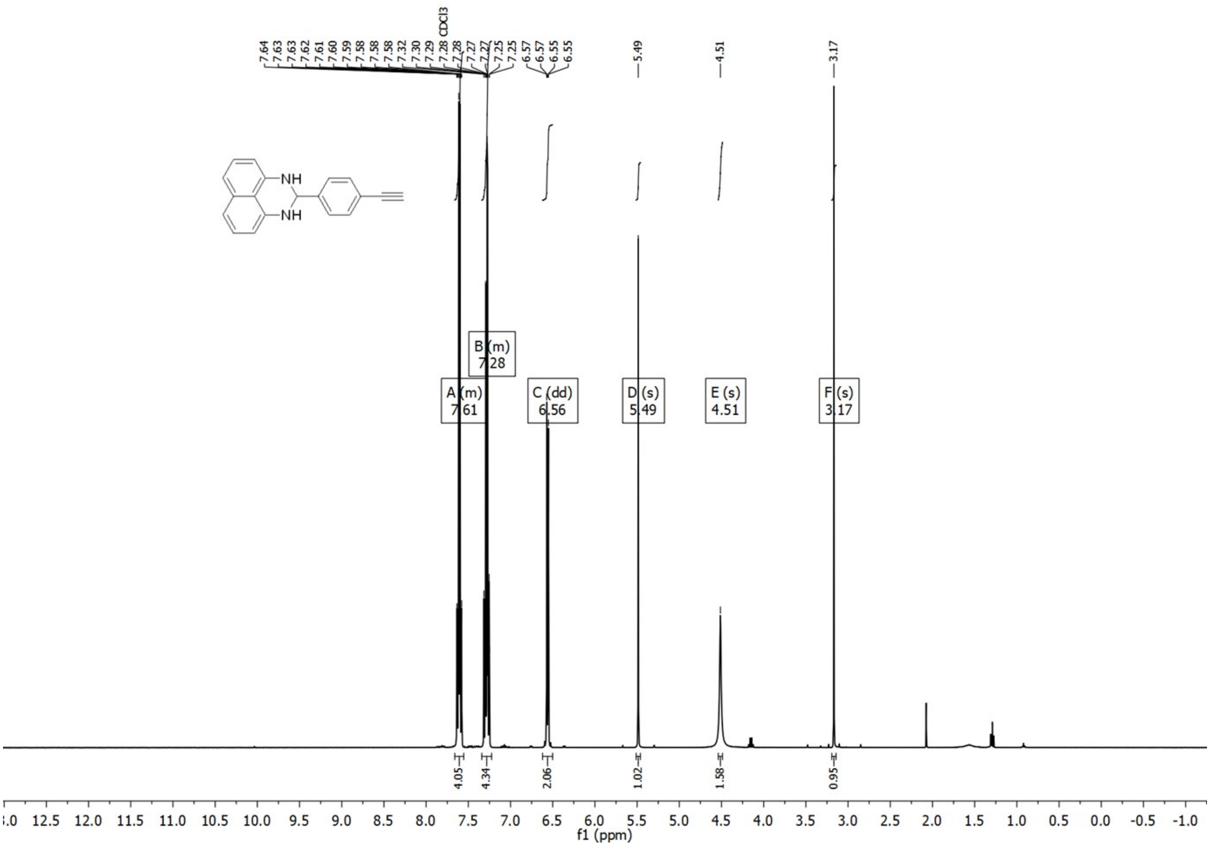

Figure 3. ^{1}H-NMR of 2-(4-Ethynylphenyl)-2,3-dihydro-1H-perimidine (**2**)

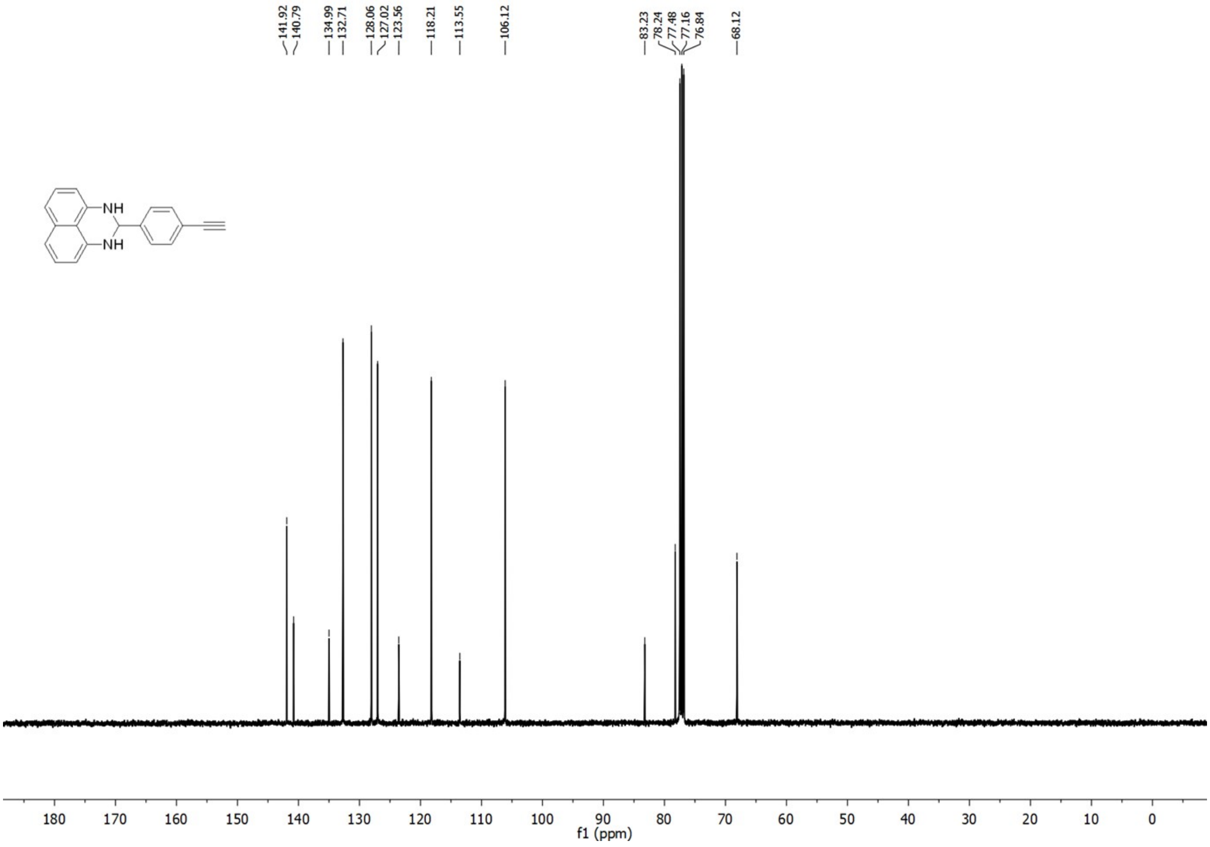

Figure 4. ^{13}C-NMR of 2-(4-Ethynylphenyl)-2,3-dihydro-1H-perimidine (**2**)

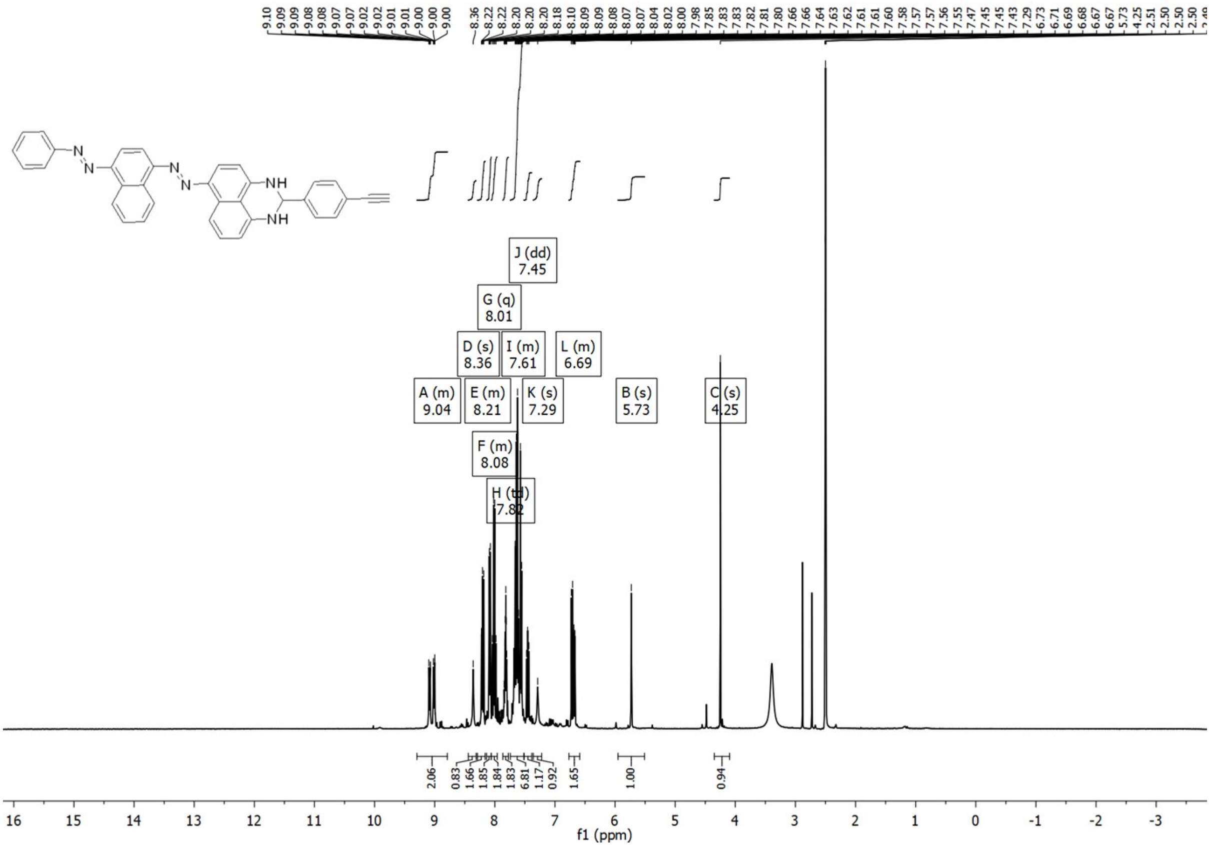

Figure 5. ^{1}H-NMR of 2-(4-ethynylphenyl)-6-((E)-(4-((E)-phenyldiazenyl)naphthalen-1-yl)diazenyl)-2,3-dihydro-1H-perimidine (**3**)

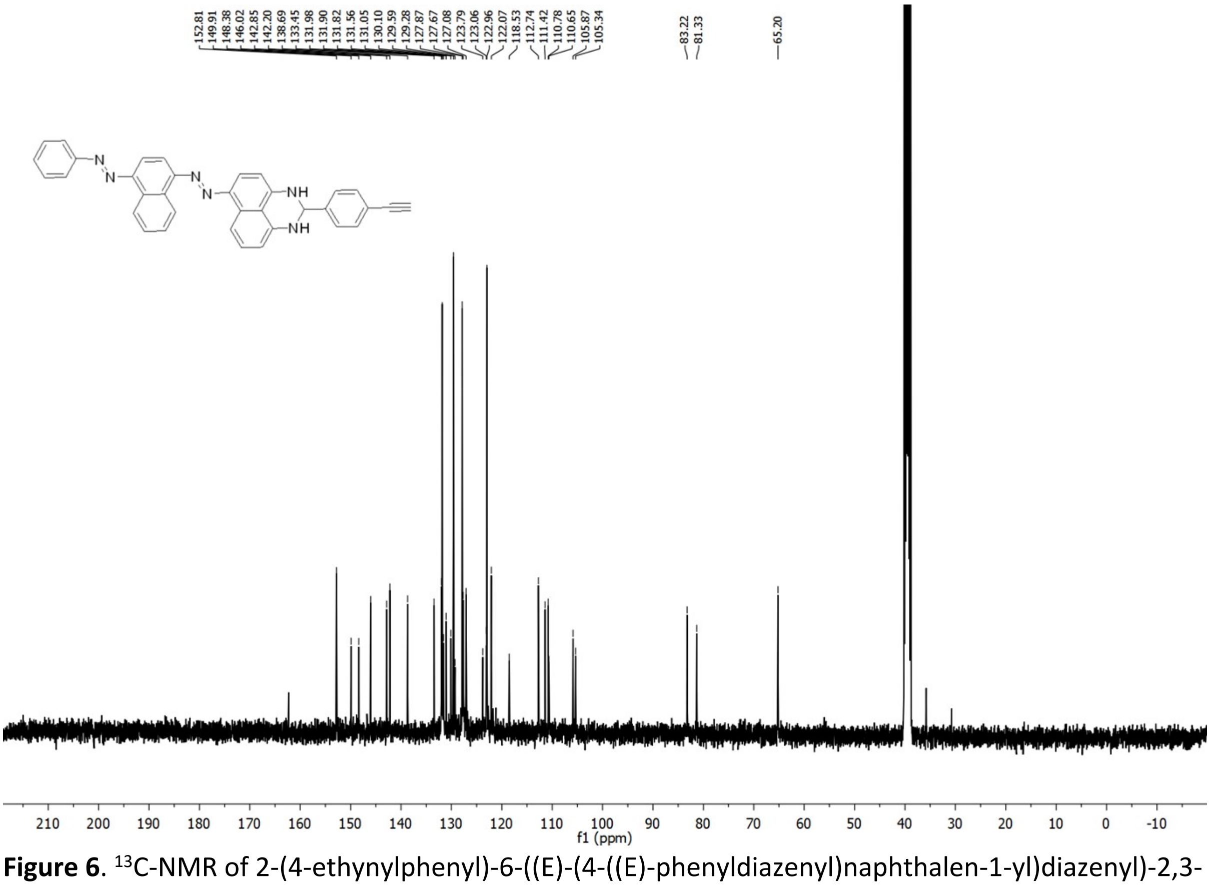

Figure 6. ^{13}C-NMR of 2-(4-ethynylphenyl)-6-((E)-(4-((E)-phenyldiazenyl)naphthalen-1-yl)diazenyl)-2,3-dihydro-1H-perimidine (**3**)

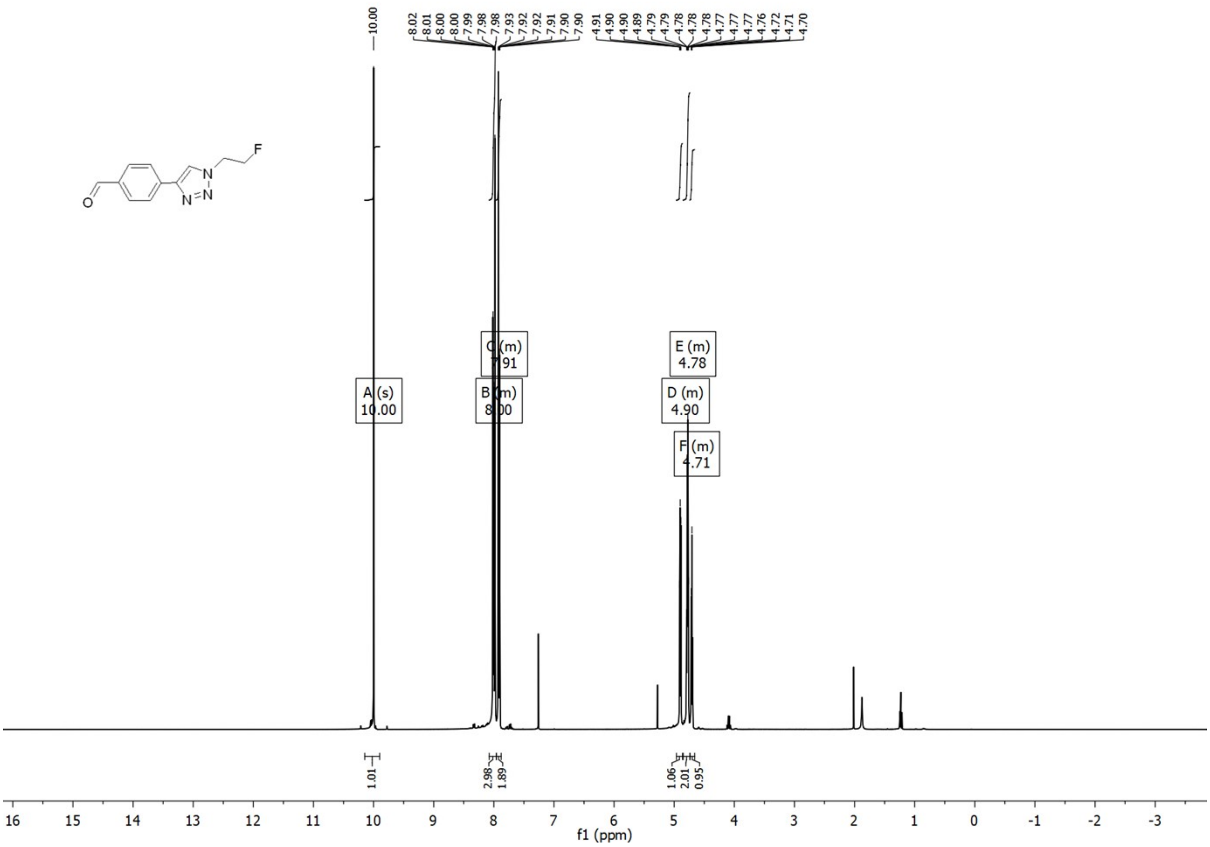

Figure 7. ^{1}H-NMR of 4-(1-(2-fluoroethyl)-1H-1,2,3-triazol-4-yl)benzaldehyde (**4**)

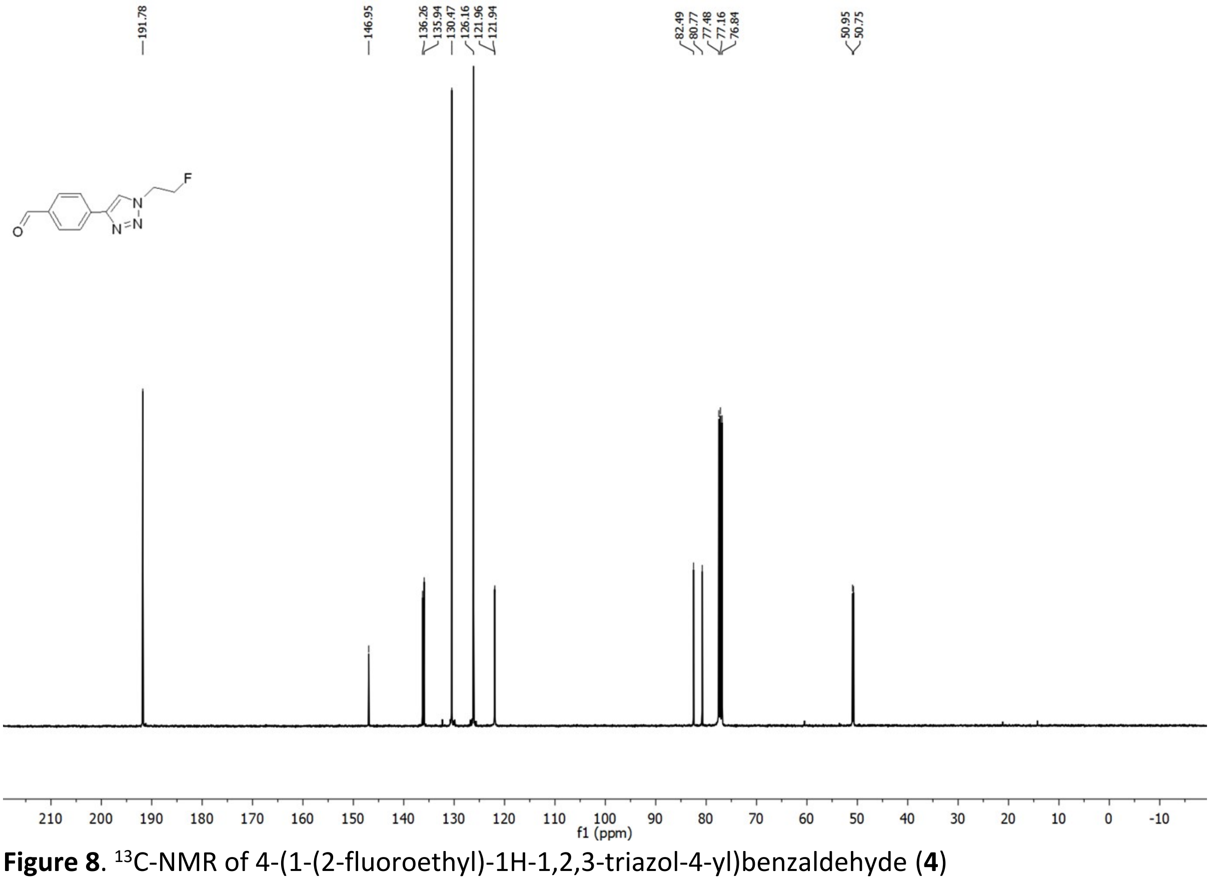

Figure 8. ^{13}C-NMR of 4-(1-(2-fluoroethyl)-1H-1,2,3-triazol-4-yl)benzaldehyde (**4**)

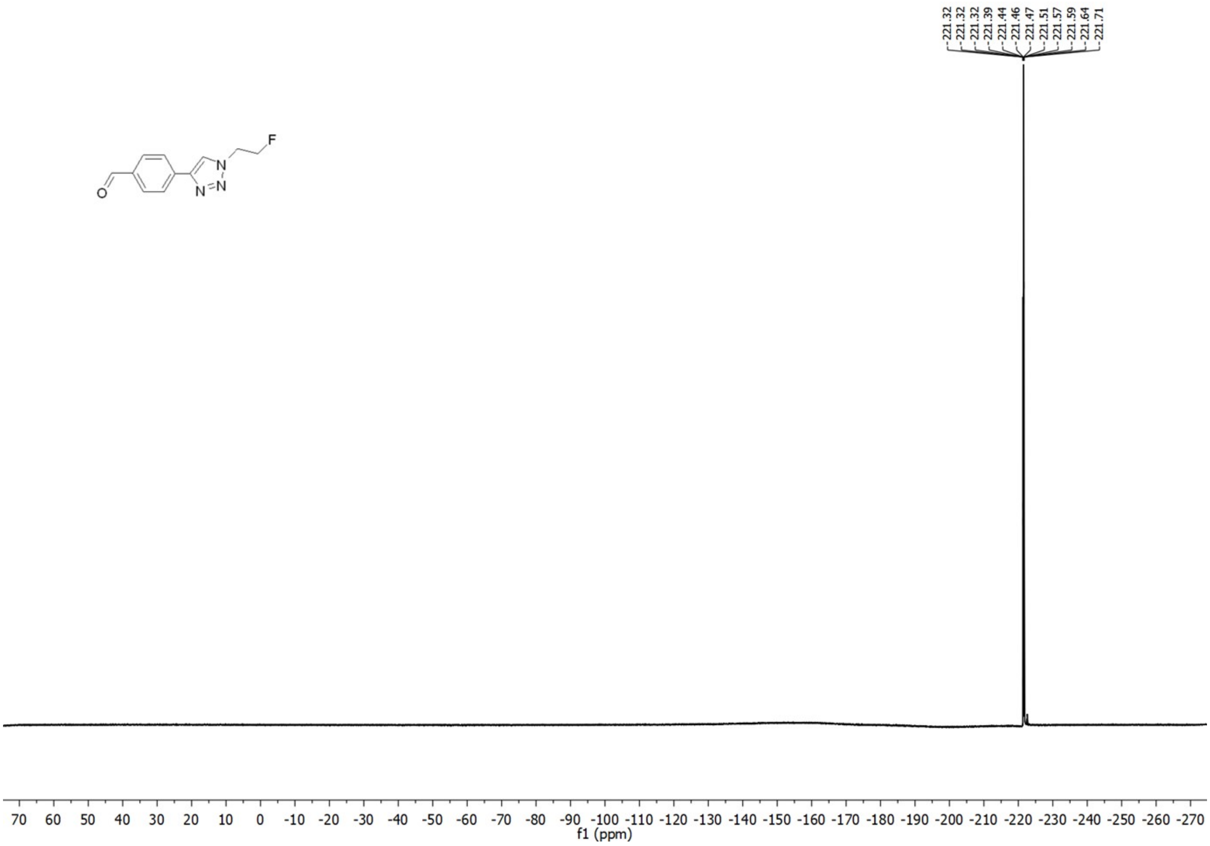

Figure 9. ^{19}F-NMR of 4-(1-(2-fluoroethyl)-1H-1,2,3-triazol-4-yl)benzaldehyde (**4**)

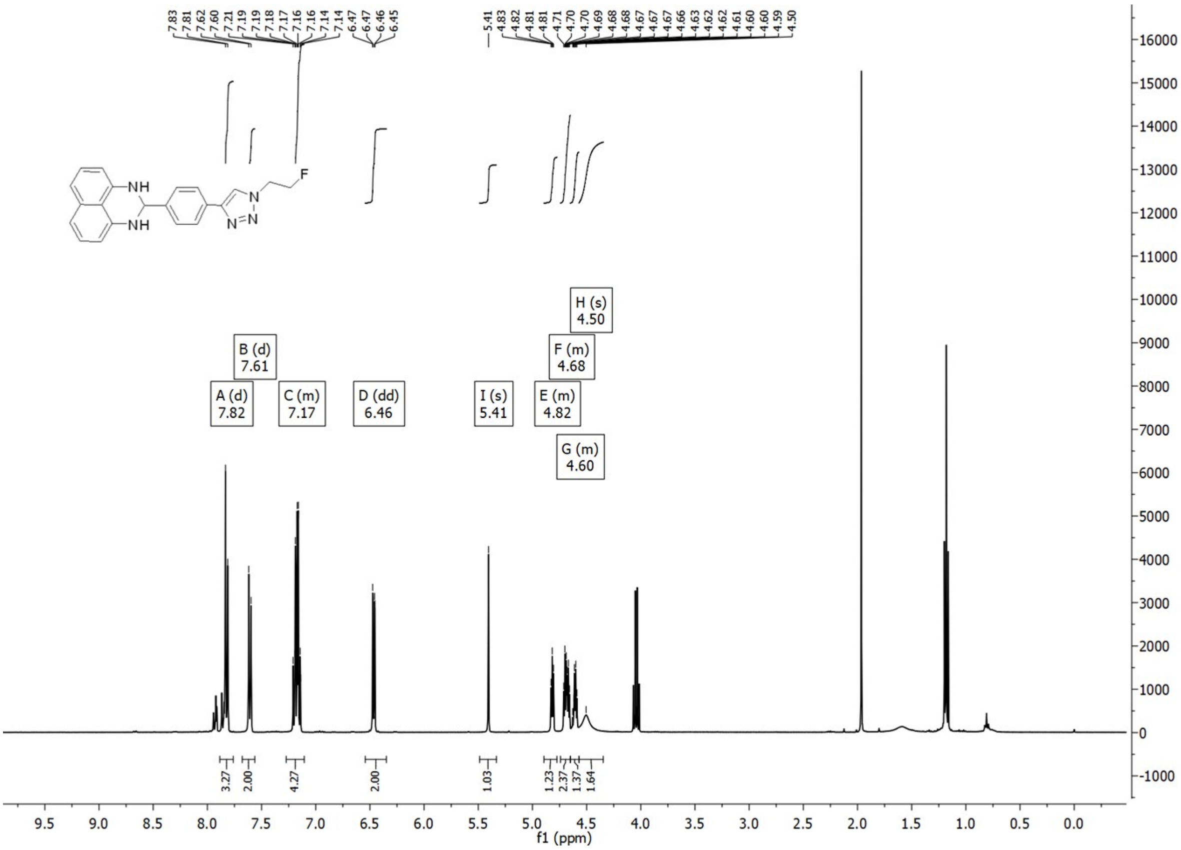

Figure 10. ^{1}H-NMR of 2-(4-(1-(2-fluoroethyl)-1H-1,2,3-triazol-4-yl)phenyl)-2,3-dihydro-1H-perimidine (**5**)

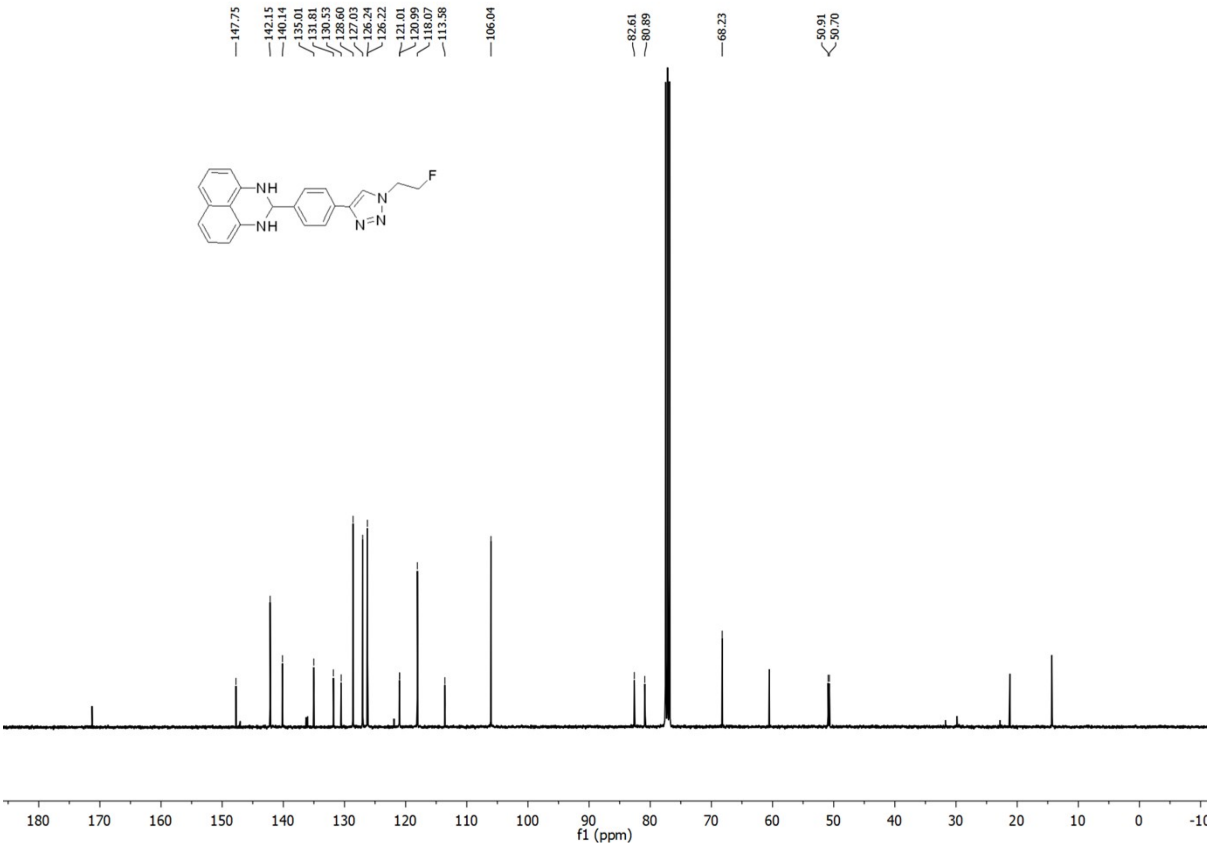

Figure 11. ^{13}C-NMR of 2-(4-(1-(2-fluoroethyl)-1H-1,2,3-triazol-4-yl)phenyl)-2,3-dihydro-1H-perimidine (**5**)

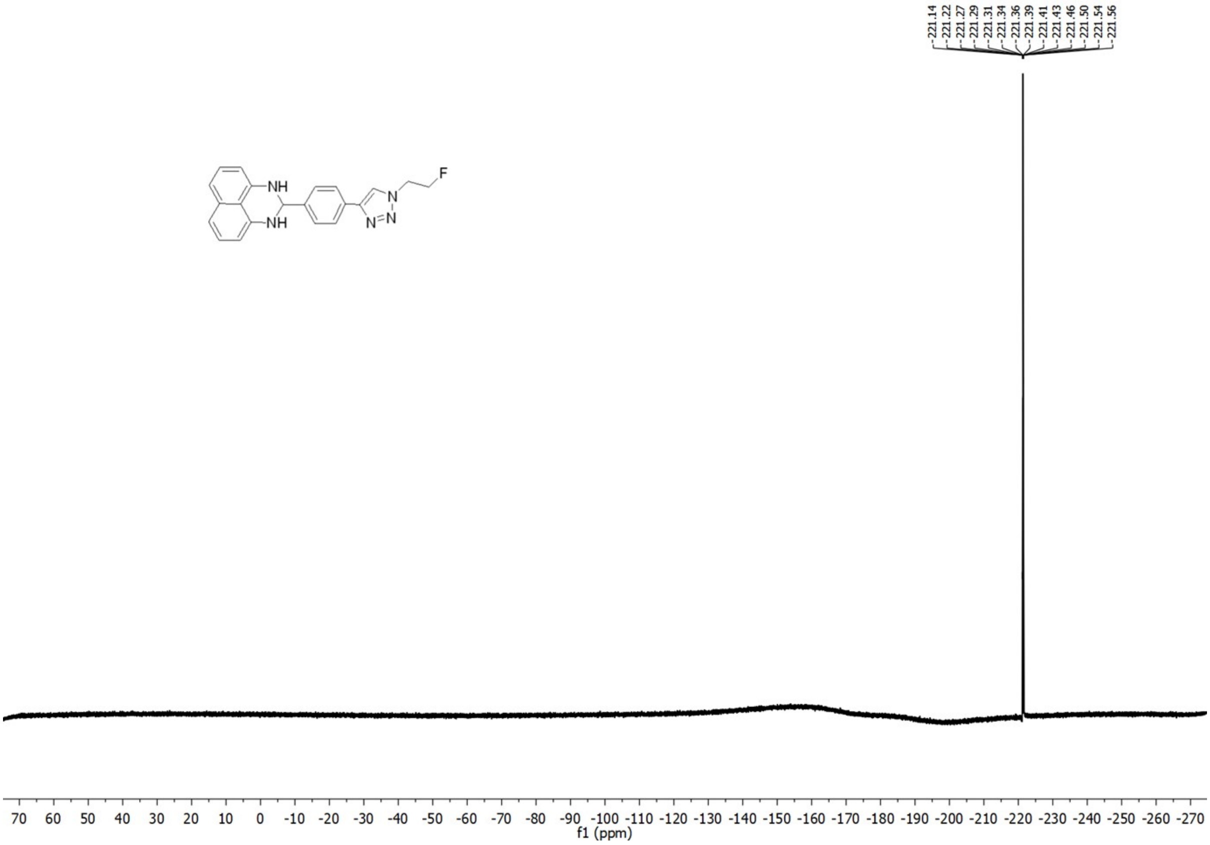

Figure 12. ^{19}F-NMR of 2-(4-(1-(2-fluoroethyl)-1H-1,2,3-triazol-4-yl)phenyl)-2,3-dihydro-1H-perimidine (**5**)

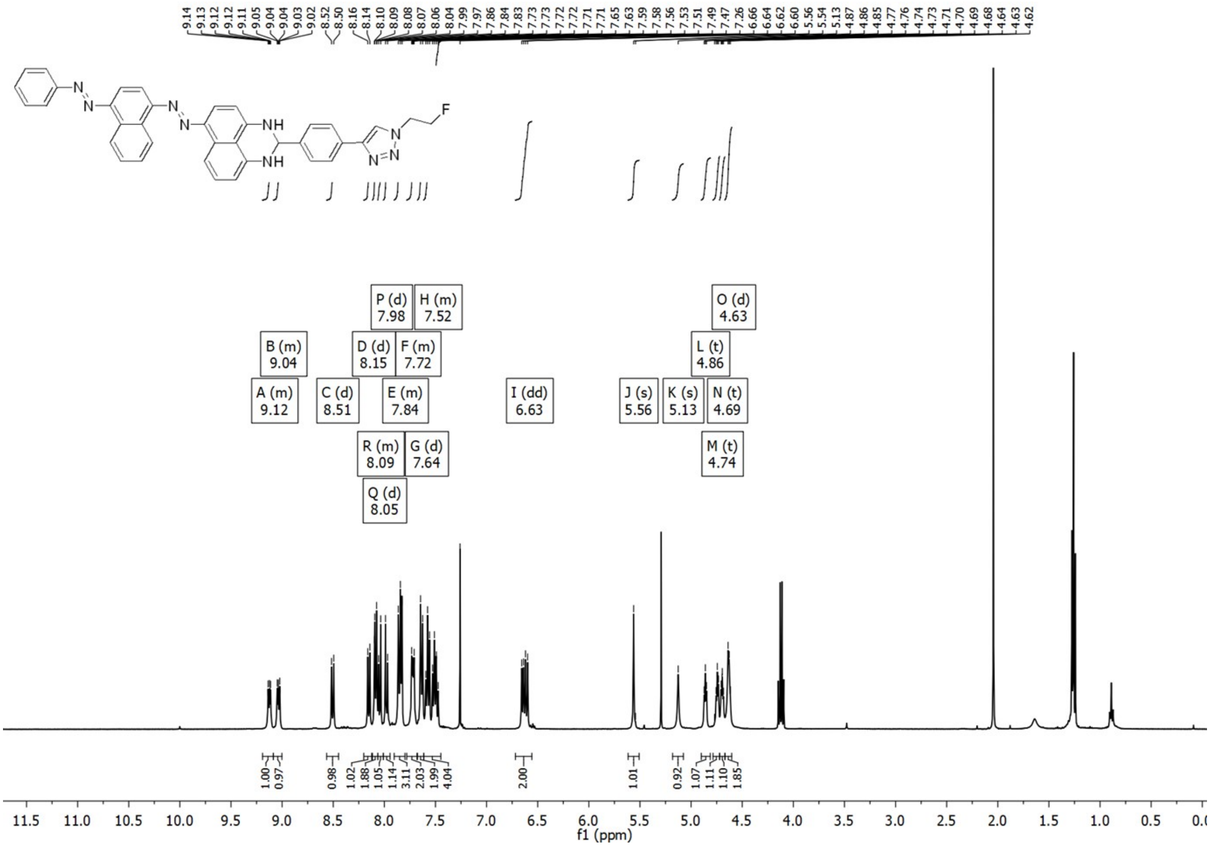

Figure 13. ^{1}H-NMR of 2-(4-(1-(2-fluoroethyl)-1H-1,2,3-triazol-4-yl)phenyl)-6-((E)-(4-((E)-phenyldiazenyl)naphthalen-1-yl)diazenyl)-2,3-dihydro-1H-perimidine (**6**)

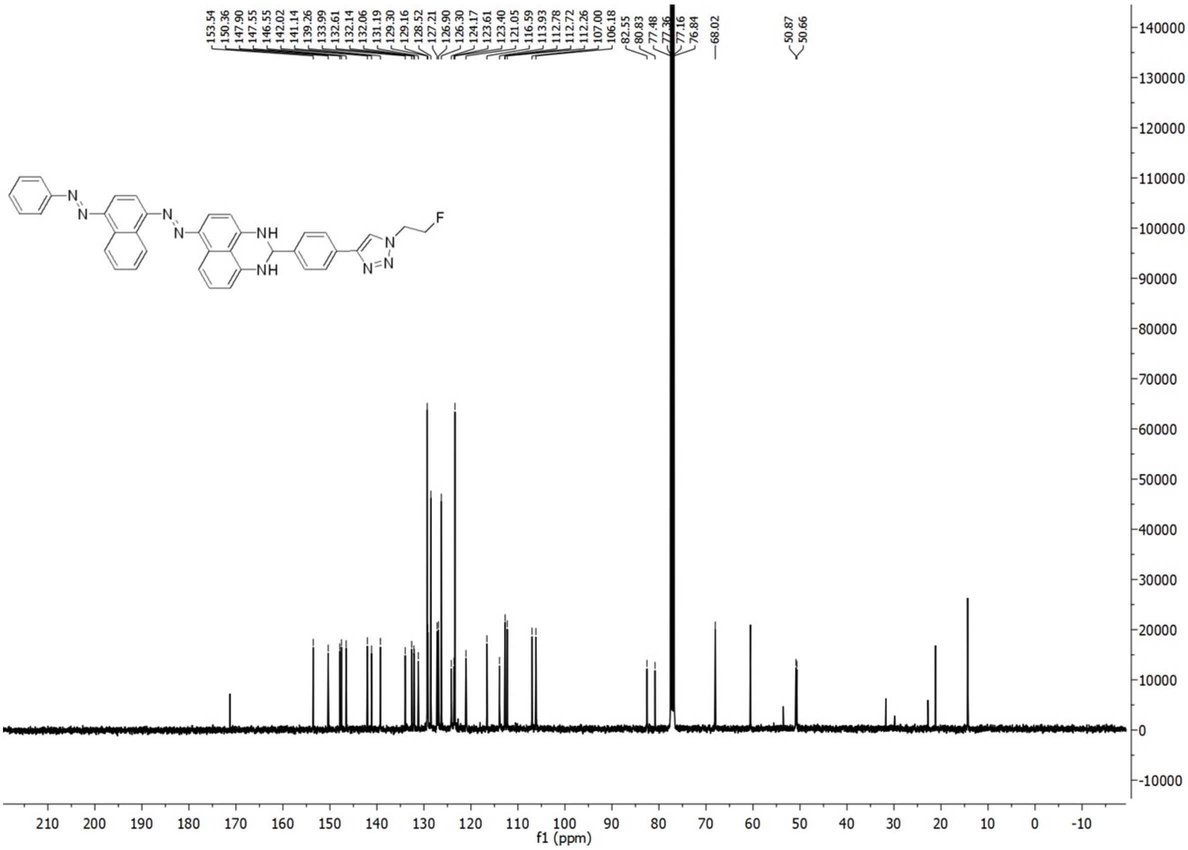

Figure 14. ^{13}C-NMR of 2-(4-(1-(2-fluoroethyl)-1H-1,2,3-triazol-4-yl)phenyl)-6-((E)-(4-((E)-phenyldiazenyl)naphthalen-1-yl)diazenyl)-2,3-dihydro-1H-perimidine (**6**)

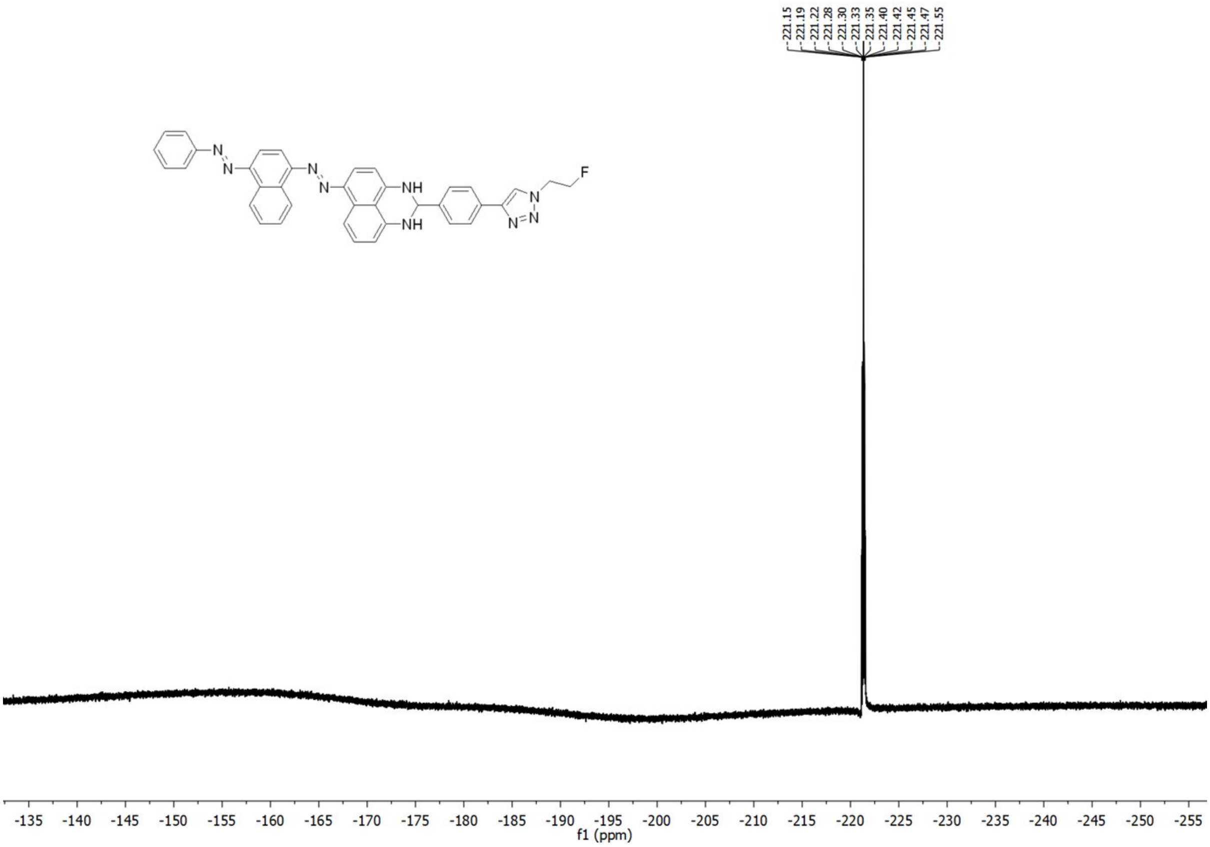

Figure 15. ^{19}F-NMR of 2-(4-(1-(2-fluoroethyl)-1H-1,2,3-triazol-4-yl)phenyl)-6-((E)-(4-((E)-phenyldiazenyl)naphthalen-1-yl)diazenyl)-2,3-dihydro-1H-perimidine (**6**)

4.0 Radiochemistry

[18F]Fluoride was produced by a GE PETtrace cyclotron by 16 MeV irradiation of enriched [18O]H_2O target, supplied by Alliance Medical Radiopharmacy Ltd (Warwick, UK). Automated radiosyntheses were performed using the GE FASTlab™ automated synthesis module (GE Healthcare Life Sciences, Amersham, UK). Solid phase extraction (SPE) cartridges were purchased from Waters (Elstree, Hertfordshire, UK) and used according to the manufacturers recommended guidelines. Semi-preparative RP-HPLC was performed using a Shimadzu LC20-AT pump attached to a custom-built system, equipped with an Agilent Eclipse XDB-C18, 5µ (250 x 9.4 mm) column. The mobile phase was 85% MeCN / 15% H_2O.

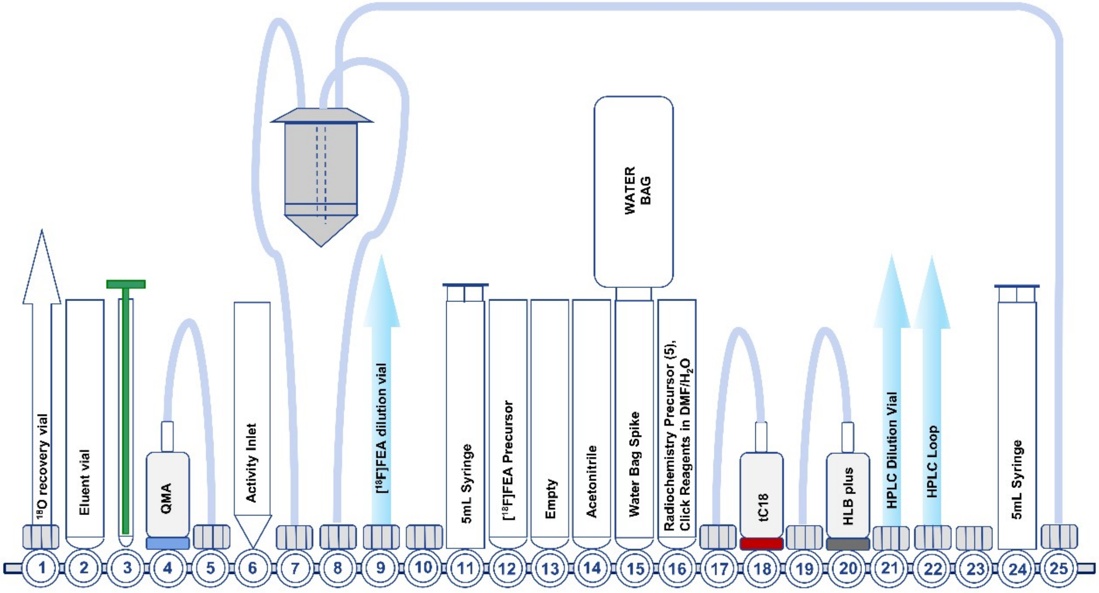

Figure 16. Schematic of the FASTLab™ cassette used to synthesise **[18F]FET-SBB**

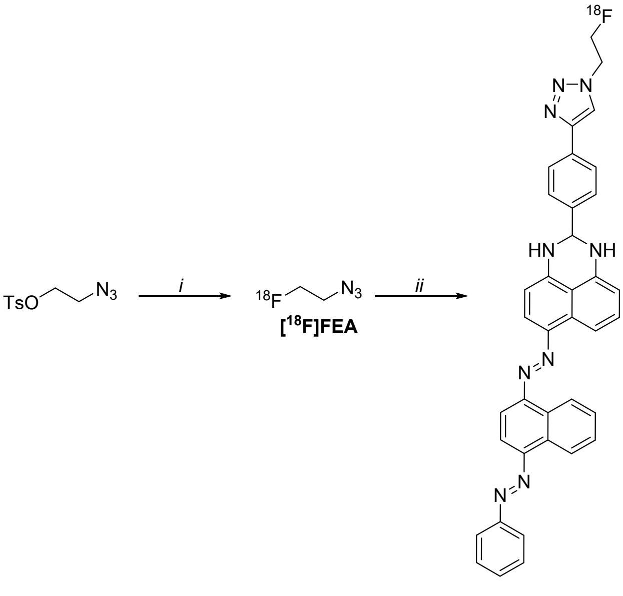

[18F]FET-SBB

Scheme 3. Radiosynthesis of **[18F]FET-SBB.** *Reaction conditions: i)* K_{222}/$KHCO_3$, MeCN, 80 °C, 10 min; *ii)* $CuSO_4.5H_2O$, Na-ascorbate, BPDS, DMF, H_2O, RT, 10 min.

5.0 HPLC Chromatograms

Reaction efficiency and radioactive product identity was determined by RP-HPLC using an Agilent 1200 series instrument connected to a flow-ram detector (Lablogic, Sheffield, UK). The system was equipped with a Phenomenex Gemini 5µ C18 110 Å (150 × 4.6 mm) column; the mobile phase was A: H_2O (0.1% TFA) and B: MeCN. The gradient was: 0 – 1 min, 70% A. 2– 22 min, 5% A. 23 –27 min, 5% A at 1 mL/min. Elution profiles were analysed using Laura software (Lablogic, Sheffield, UK).

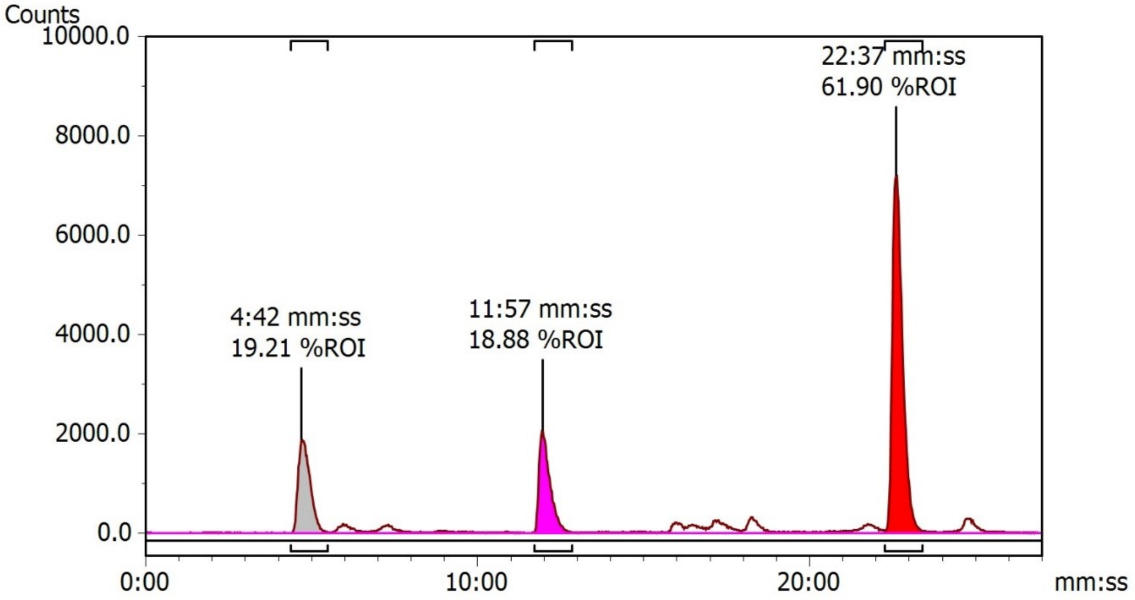

Figure 17. Representative HPLC chromatogram showing the crude radiolabelling reaction mixture; **[18F]FEA** (t_R = 4:43 mm:ss), an unknown radioactive impurity (t_R = 11:57 mm:ss) and **[18F]FET-SBB** (t_R = 22:37)

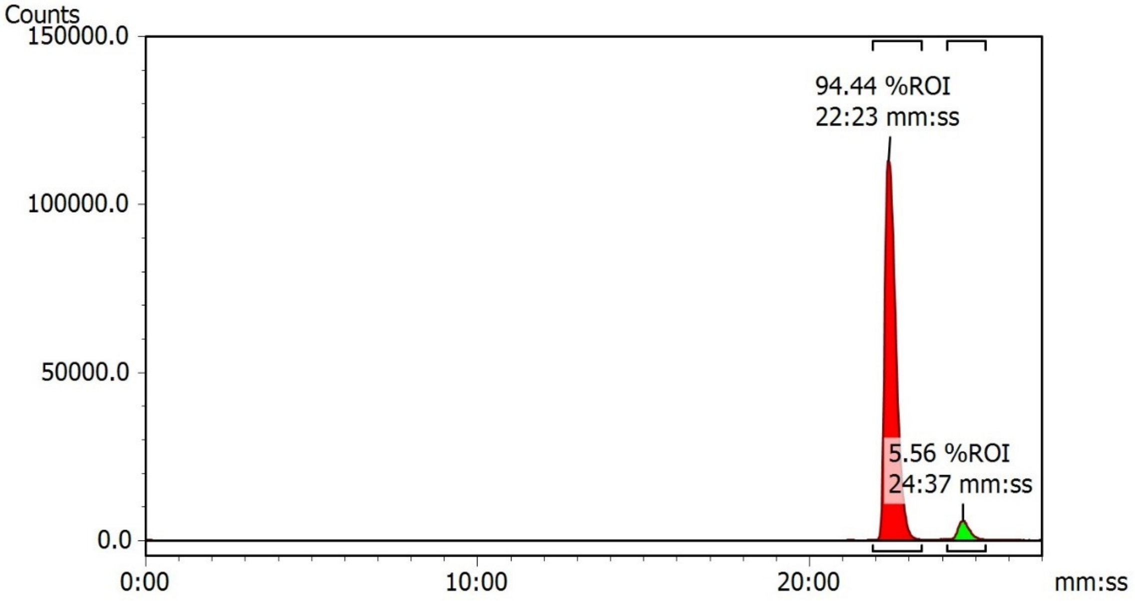

Figure 18. Representative HPLC chromatogram showing purified **[18F]FET-SBB** (t_R = 22:23 mm:ss)

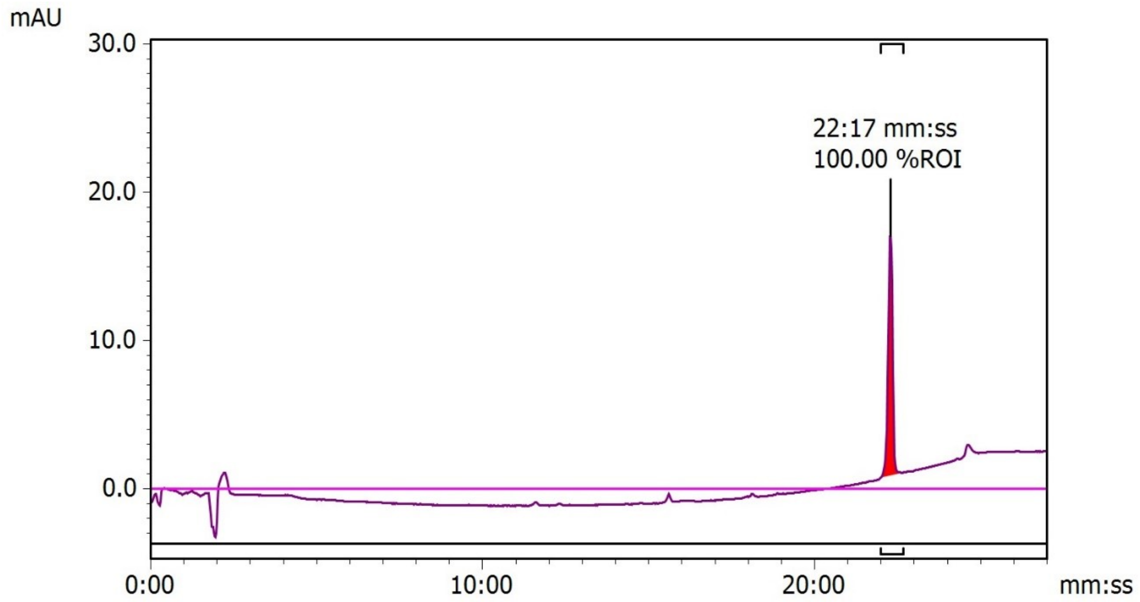

Figure 19. [^{19}F]FET-SBB (t_R = 22:17 mm:ss) reference standard (1 µg/mL)

6.0 Biological Evaluation

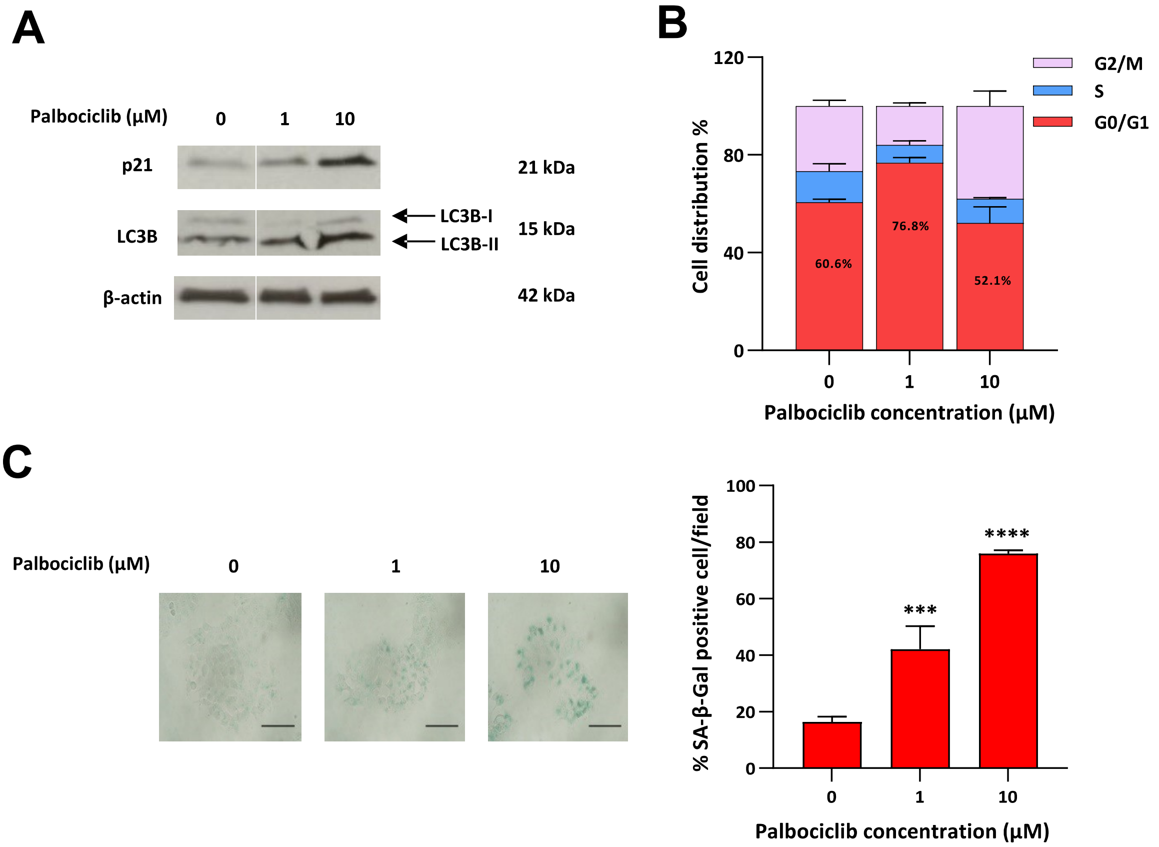

Figure 20. A) Western blot analysis of the levels of p21 and LC3B 72 h following Palbociclib treatment (1 and 10 µM) in MCF-7 cells. β-actin was used as a loading control. **B)** Flow cytometric analysis of palbociclib-induced cell cycle arrest in MCF-7 cells. Mean values ± SD (n=3). **C)** Representative images and quantification of SA-β-gal staining in MCF-7 cells following Palbociclib treatment. *** $p < 0.001$ and **** $p < 0.0001$ indicate significant difference from the value measured in control untreated group. Scale bar = 50 µm.

7.0 PET Imaging Data

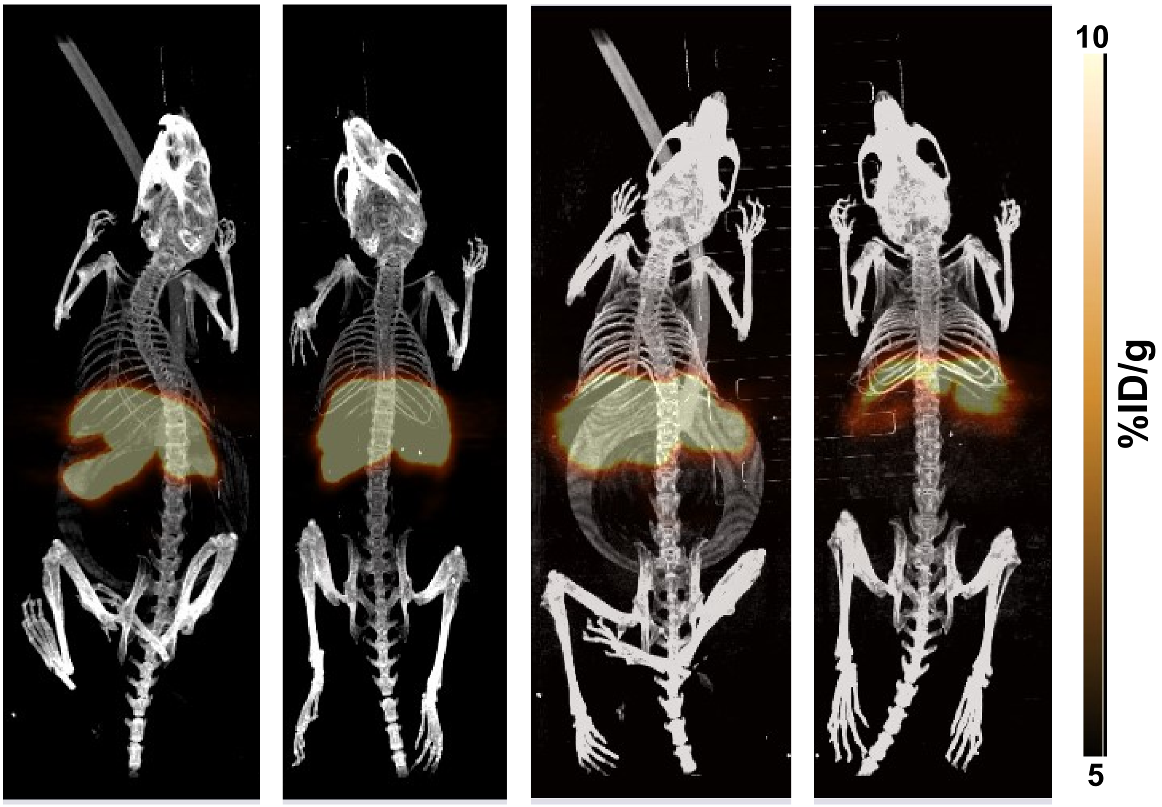

Figure 21. *In vivo* PET imaging of [^{18}F]FET-SBB in four mice. Maximum intensity projection images are presented (30 – 60 min) after injection of 2 MBq of [^{18}F]FET-SBB *via* the tail vein.
